# Supplementary material for: Pharmacological Activation of Non-canonical NF-κB Signaling Activates Latent HIV-1 Reservoirs In Vivo
Source: Cell Rep Med. 2020 Jun 23;1(3):100037. doi: 10.1016/j.xcrm.2020.100037 (PMC7659604; doi:10.1016/j.xcrm.2020.100037)
Supplement: Document S1. Figures S1–S3 and Table S1 [file mmc1.pdf]

Cell Reports Medicine, Volume *1*

Supplemental Information

Pharmacological Activation of Non-canonical NF-κB Signaling Activates Latent HIV-1 Reservoirs *In Vivo*

Lars Pache, Matthew D. Marsden, Peter Teriete, Alex J. Portillo, Dominik Heimann, Jocelyn T. Kim, Mohamed S.A. Soliman, Melanie Dimapasoc, Camille Carmona, Maria Celeridad, Adam M. Spivak, Vicente Planelles, Nicholas D.P. Cosford, Jerome A. Zack, and Sumit K. Chanda

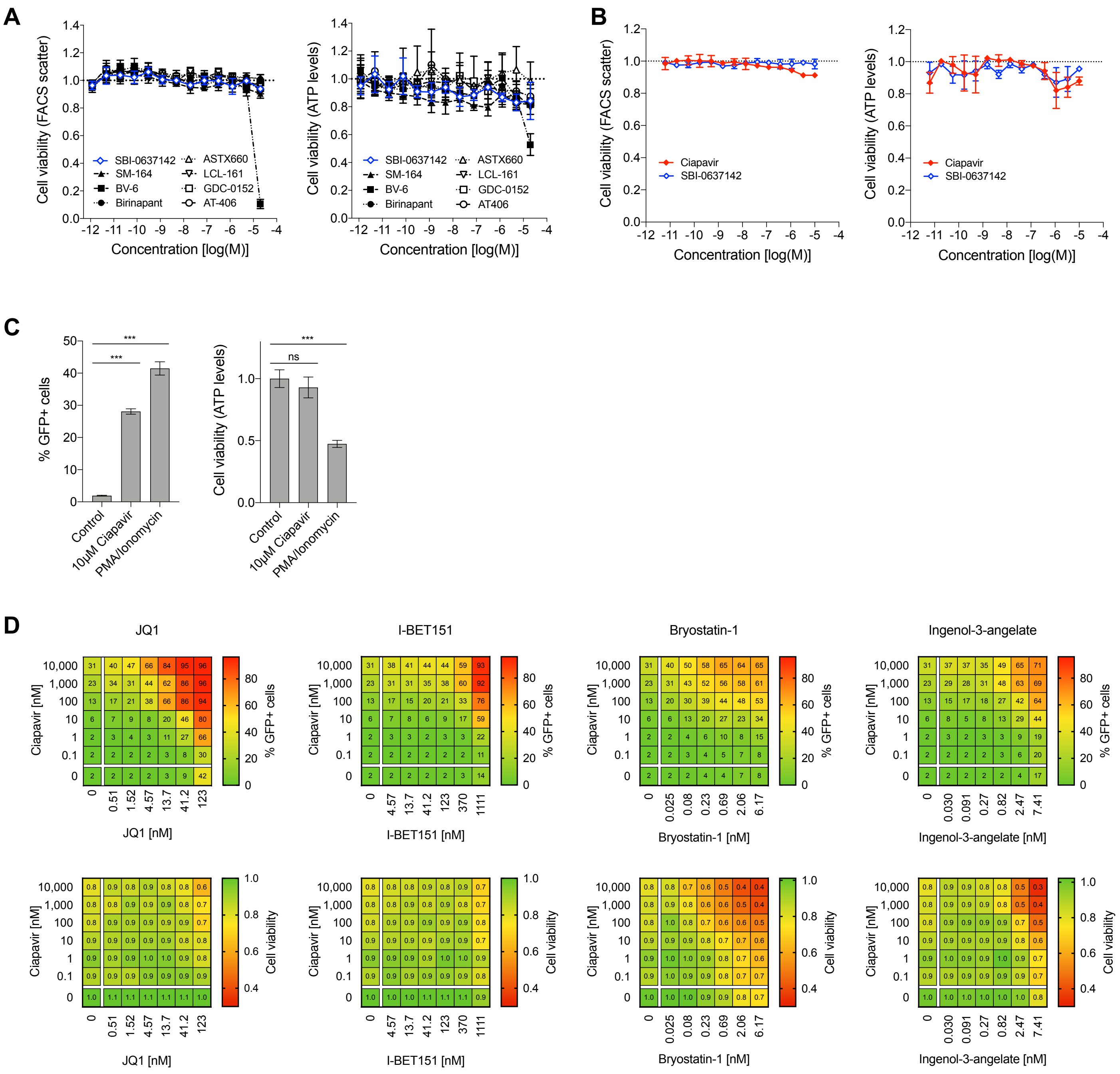

Supplemental Figure 1. Ciapavir is not toxic in 2D10 cells and synergizes with JQ1 and I-BET151. Related to Figure 1. **A, B)** Cell viability of 2D10 cells upon Smac mimetic treatment was assessed after 48 h by flow cytometry scatter analysis or by measuring cellular ATP levels. Data corresponds to Figures 1B and 1E, respectively, and was normalized to mock-treated samples. Data represent mean and SD (n = 2 (A); n = 3 (B)). **(C)** 2D10 cells were treated with 10 µM Ciapavir, 50 ng/ml PMA and 1 µM ionomycin, or DMSO as negative control for 48 h prior to analysis by flow cytometry. Cell viability was determined by measuring cellular ATP levels and normalized to DMSO-treated negative control. Data represent mean and SD of three biological replicates. Significance was determined with a t-test (n = 3). **D)** Ciapavir acts synergistically with JQ1 and I-BET151. 2D10 cells were treated with combinations of Ciapavir and JQ1, I-BET151, bryostatin-1, or ingenol-3-angelate for 48h and analyzed by flow cytometry for GFP expression. Cell viability was analyzed by measuring cellular ATP levels. Heatmaps show averaged data of two experiments.

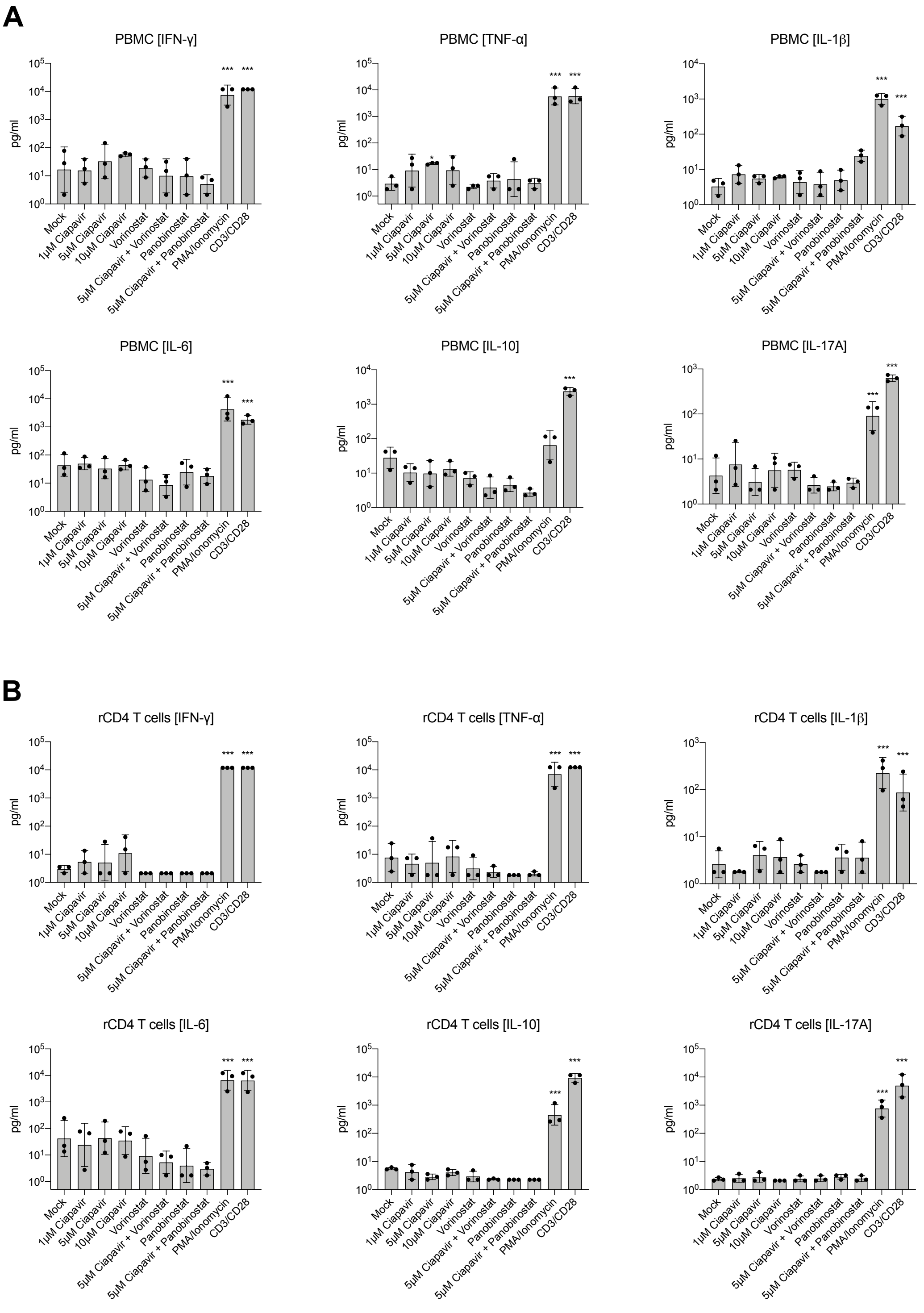

Supplemental Figure 2. Ciapavir does not induce cytokine release in human PBMC or rCD4^{+} T cells. Related to Figure 2. Values of individual donors corresponding to the data represented in Figures 2A and 2B are shown in **A)** and **B)**, respectively. Bars represent geometric mean and geometric standard deviation of three donors. To determine significance of cytokine upregulation, log transformed data was analyzed with a 2-way ANOVA using Dunnett's multiple comparison correction (n = 3).

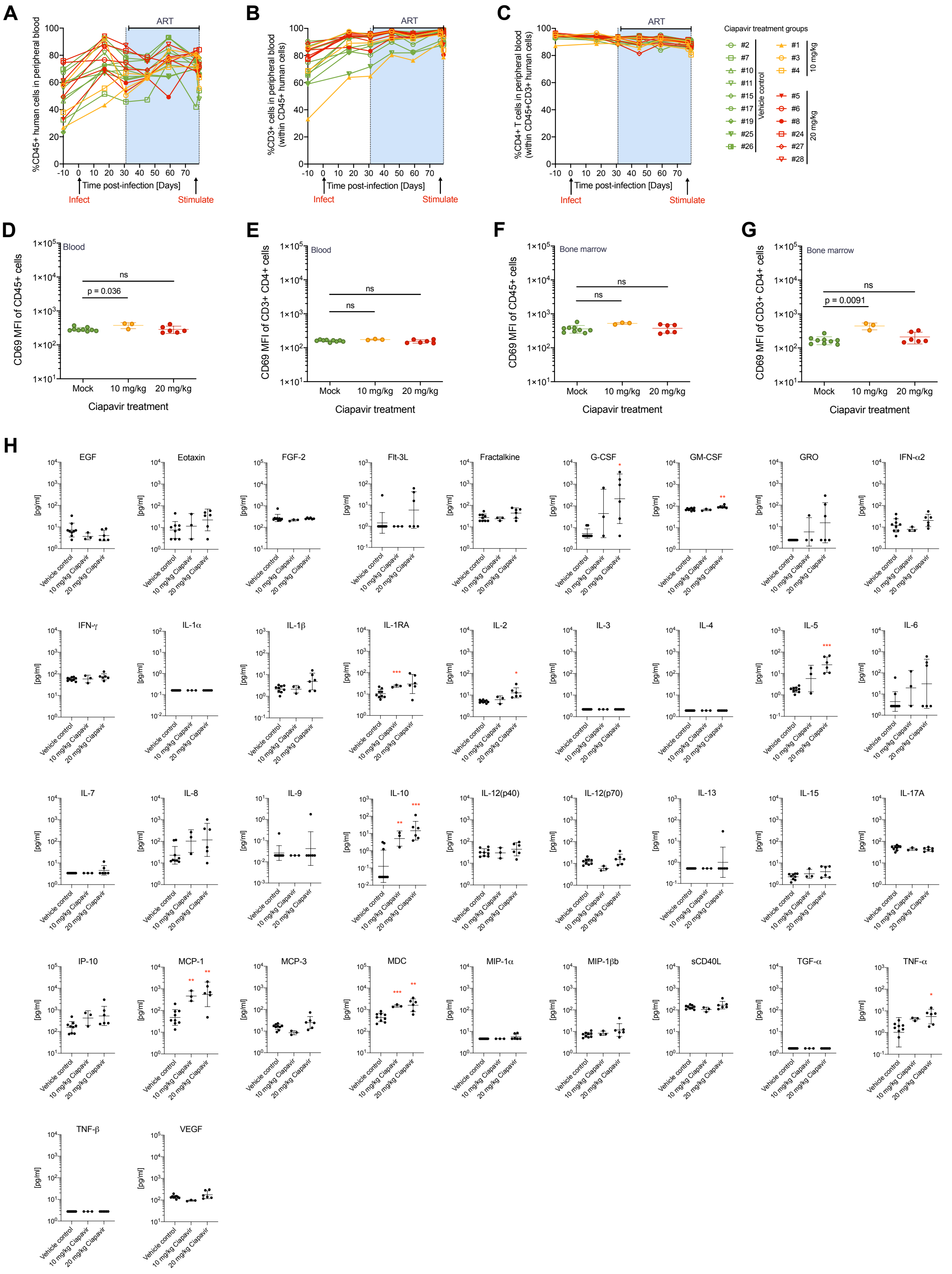

Supplemental Figure 3. Limited *in vivo* immune activation following Smac mimetic administration to humanized mice. Related to Figure 4. The frequencies of **(A)** overall human $CD45^{+}$ immune cells, **(B)** human T cells ($CD45^{+}CD3^{+}$), and **(C)** human $CD4^{+}$ T cells ($CD45^{+}CD3^{+}CD4^{+}$), analyzed by flow cytometry, did not differ significantly between treatment groups at any timepoint ($p > 0.05$, 2-sided, unpaired, equal variance t-test). Panels B and C represent frequencies of the populations from their parent gates. Mean fluorescence intensities (MFI) of samples analyzed in Figure 4E-H are shown for all blood $CD45^{+}$ cells **(D)** and $CD4^{+}$ T cells **(E)** as well as bone marrow $CD45^{+}$ **(F)** and $CD4^{+}$ T cells **(G)**. Error bars represent mean and SD ($n = 9$ for mock, $n = 3$ for 10 mg/kg Ciapavir, $n = 6$ for 20 mg/kg Ciapavir). Statistics represent 2-sided Mann-Whitney test. **H)** Plasma cytokine levels in BLT mice were analyzed 48 h post treatment. Data represent geometric mean and geometric SD. Significance was determined by 2-way ANOVA of log transformed data using Dunnett's multiple comparison correction ($n = 9$ for mock, $n = 3$ for 10 mg/kg Ciapavir, $n = 6$ for 20 mg/kg Ciapavir).

Parameter	Unit	Value
Lambda_z	1/h	0.24
$t_{1/2}$	h	2.9
T_{max}	h	0.25
C_{max}	µmol/L	8.1
T_{lag}	h	0.00
C_{last_obs}/C_{max}		0.00
AUC $_{0-t}$	µmol/L*h	10.2
AUC $_{0-inf\_obs}$	µmol/L*h	10.2
AUC $_{0-t/0-inf\_obs}$		1.0
AUMC $_{0-inf\_obs}$	µmol/L*h^2	15.8
MRT $_{0-inf\_obs}$	h	1.5
Vz/F_obs	(mg/kg)/(µmol/L)	4.1
Cl/F_obs	(mg/kg)/(µmol/L)/h	0.98

Supplemental Table 1: Detailed pharmacokinetic parameters. Related to Figure 3.
