# Supplementary material for: Pharmacological Activation of Non-canonical NF-κB Signaling Activates Latent HIV-1 Reservoirs In Vivo
Source: Cell Rep Med. 2020 Jun 23;1(3):100037. doi: 10.1016/j.xcrm.2020.100037 (PMC7659604; doi:10.1016/j.xcrm.2020.100037)
Supplement: Document S2. Article plus Supplemental Information [file mmc2.pdf]

Cell Reports
Medicine

Report

Pharmacological Activation of Non-canonical NF-κB Signaling Activates Latent HIV-1 Reservoirs *In Vivo*

Graphical Abstract

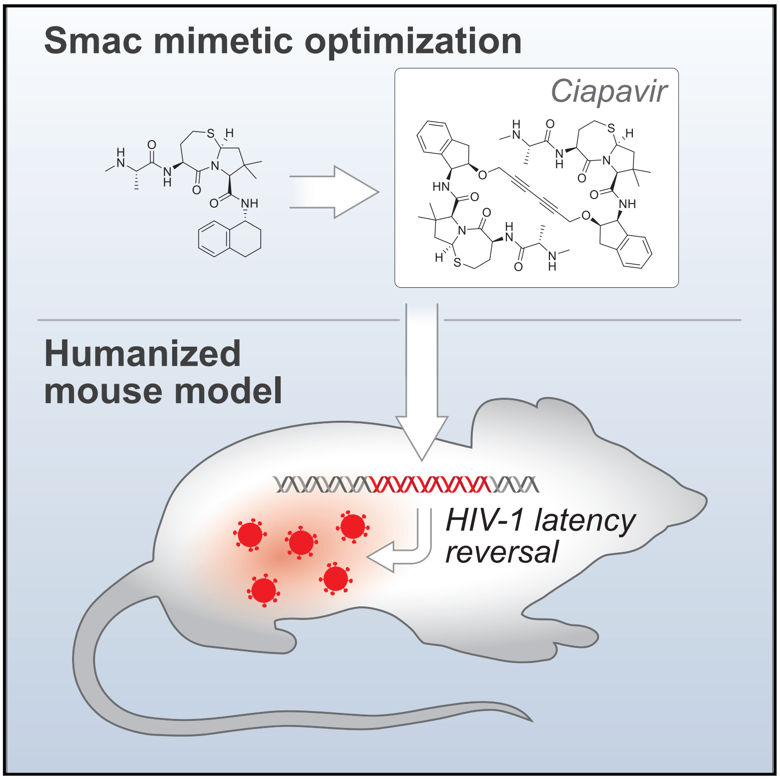

Highlights

- Ciapavir is a potent Smac mimetic specifically optimized for HIV-1 latency reversal
- Smac mimetics synergize with bromodomain inhibitors to reverse HIV-1 latency
- Ciapavir is well tolerated and does not induce broad immune activation
- Systemic administration of Ciapavir mediates latency reversal in a mouse model

Authors

Lars Pache, Matthew D. Marsden, Peter Teriete, ..., Nicholas D.P. Cosford, Jerome A. Zack, Sumit K. Chanda

Correspondence

schanda@sbpdiscovery.org

In Brief

Pache et al. report the development of Ciapavir, a potent small-molecule Smac mimetic optimized for HIV-1 latency reversal. Ciapavir shows favorable pharmacokinetic and pharmacodynamic properties in mice and induces activation of the latent HIV-1 reservoir *in vivo* in a humanized mouse model in the absence of systemic immune activation.

Pache et al., 2020, Cell Reports Medicine *1*, 100037
June 23, 2020 © 2020 The Author(s).
https://doi.org/10.1016/j.xcrm.2020.100037

CellPress

Cell Reports Medicine

CellPress
OPEN ACCESS

Report

Pharmacological Activation of Non-canonical NF-κB Signaling Activates Latent HIV-1 Reservoirs *In Vivo*

Lars Pache,[1] Matthew D. Marsden,[2,7] Peter Teriete,[3,7] Alex J. Portillo,[1] Dominik Heimann,[3] Jocelyn T. Kim,[4] Mohamed S.A. Soliman,[5] Melanie Dimapasoc,[5] Camille Carmona,[5] Maria Celeridad,[3] Adam M. Spivak,[6] Vicente Planelles,[6] Nicholas D.P. Cosford,[3,8] Jerome A. Zack,[2,5,8] and Sumit K. Chanda[1,8,9,*]

[1]Infectious and Inflammatory Disease Center, Immunity and Pathogenesis Program, Sanford Burnham Prebys Medical Discovery Institute, La Jolla, CA 92037, USA

[2]Division of Hematology and Oncology, Department of Medicine, University of California, Los Angeles, Los Angeles, CA 90095, USA

[3]Cell Metabolism and Signaling Networks Program, NCI-Designated Cancer Center, Sanford Burnham Prebys Medical Discovery Institute, La Jolla, CA 92037, USA

[4]Division of Infectious Diseases, Department of Medicine, University of California, Los Angeles, Los Angeles, CA 90095, USA

[5]Department of Microbiology, Immunology, and Molecular Genetics, University of California, Los Angeles, Los Angeles, CA 90095, USA

[6]Division of Microbiology and Immunology, Department of Pathology, University of Utah School of Medicine, Salt Lake City, UT 84112, USA

[7]These authors contributed equally

[8]Senior author

[9]Lead Contact

*Correspondence: schanda@sbpdiscovery.org

https://doi.org/10.1016/j.xcrm.2020.100037

SUMMARY

“Shock and kill” strategies focus on purging the latent HIV-1 reservoir by treating infected individuals with therapeutics that activate the latent virus and subsequently eliminating infected cells. We have previously reported that induction of non-canonical nuclear factor κB (NF-κB) signaling through a class of small-molecule antagonists known as Smac mimetics can reverse HIV-1 latency. Here, we describe the development of Ciapavir (SBI-0953294), a molecule specifically optimized for HIV-1 latency reversal that was found to be more efficacious as a latency-reversing agent than other Smac mimetics under clinical development for cancer. Critically, this molecule induced activation of HIV-1 reservoirs *in vivo* in a bone marrow, liver, thymus (BLT) humanized mouse model without mediating systemic T cell activation. This study provides proof of concept for the *in vivo* efficacy and safety of Ciapavir and indicates that Smac mimetics can constitute a critical component of a safe and efficacious treatment strategy to eliminate the latent HIV-1 reservoir.

INTRODUCTION

The development of combination antiretroviral therapy (ART) has enabled the suppression of HIV-1 replication to undetectable levels. However, the existence of latent viral reservoirs, persisting for decades, can lead to renewed viremia upon treatment interruption.[1] “Shock and kill” approaches aim to deplete the latent reservoir by treating patients with therapeutics that mediate latency reversal and the subsequent elimination of infected cells.[2] The development of safe, potent, and effective latency-reversing agents (LRAs) is an important step that would disencumber HIV-1-infected patients of life-long ART regimens. To achieve this goal, LRAs seek to safely decloak the small but persistent reservoir of latently infected cells. However, due to adverse effects or a reported lack of efficacy of LRAs explored to date, the therapeutic approaches that will constitute an effective shock therapy are currently unclear.[3] Although histone deacetylase inhibitors (HDACis) were evaluated in multiple clinical trials, only modest clinical efficacy has been reported to date.[4–8] Among the most effective LRAs *in vitro* are protein kinase C (PKC) agonists, which include bryostatin and ingenol.[9] However, this class of compounds is associated with systemic T cell activation,[10] and adverse effects have been reported in clinical trials.[11] Additionally, a recent study found that blockade of checkpoint protein programmed death-1 (PD-1) using the antibody nivolumab and activation of Toll-like receptor 7 (TLR7) with the agonist vesatolimod, previously proposed as latency-reversing treatment,[12–14] did not impact viral rebound kinetics following ART interruption in simian immunodeficiency virus (SIV)-infected macaques.[15] Therefore, a safe and effective modality for HIV-1 latency reversal continues to represent a critical unmet therapeutic need.

The inhibitor of apoptosis (IAP) protein family is a functionally and structurally related group of proteins that primarily serve as cellular inhibitors of programmed cell death, or apoptosis.[16,17] Smac mimetics are a class of small-molecule peptidomimetics derived from a conserved binding motif of Smac (second mitochondria-derived activator of caspases), an endogenous

This is an open access article under the CC BY license (http://creativecommons.org/licenses/by/4.0/).

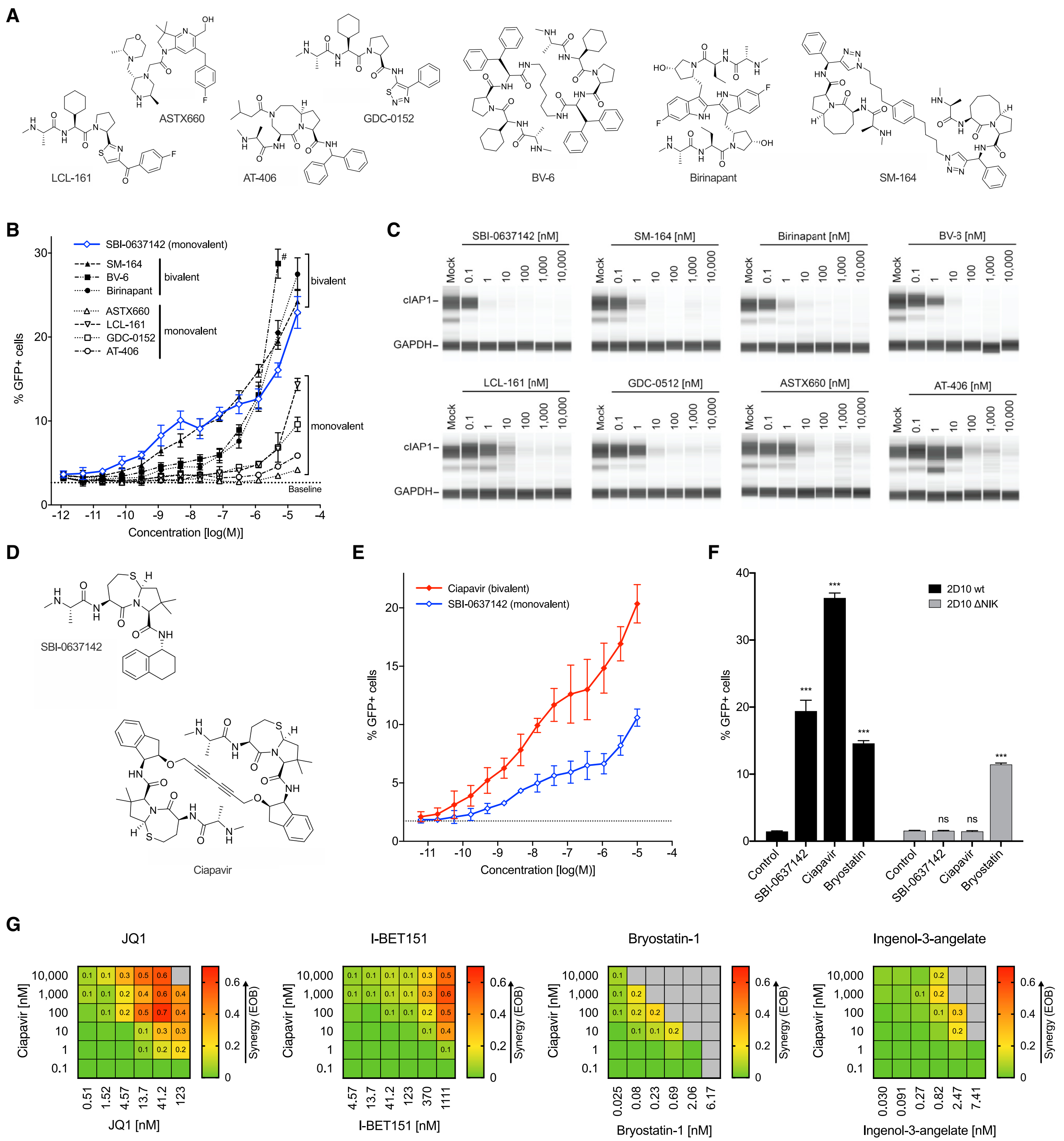

Figure 1. Ciapavir Reverses HIV-1 Latency with Improved Potency and Efficacy

(A) Structures of seven commercially available Smac mimetics that were tested for LRA activity.

(B) 2D10 Jurkat cells were incubated with SBI-0637142 and seven additional Smac mimetic compounds for 48 h. Latency reversal was assessed by measuring GFP expression by flow cytometry. Baseline activation levels are indicated by dashed line. Data represent mean and SD of two biological replicates (n = 2). # BV-6 could not be assessed at a concentration of 20 μM due to cytotoxicity (see Figure S1A).

(C) cIAP1 degradation after Smac mimetic treatment of 2D10 cells for 24 h was evaluated by automated capillary western blot (SimpleWestern) analysis.

(D) Structure of the monovalent compound SBI-0637142 and the optimized bivalent Smac mimetic Ciapavir.

(E) 2D10 cells were incubated with increasing concentrations of SBI-0637142 and Ciapavir for 48 h. Data represent mean and SD of three experiments. Difference between compound LRA activities is significant at concentrations greater than 0.5 nM ($p < 0.05$; determined by two-way ANOVA; n = 3).

(legend continued on next page)

protein inhibitor of IAPs, which include XIAP, cIAP1, cIAP2, ILP2, BRUCE/Apollon, survivin, NAIP, and ML-IAP.[18–23] Smac mimetics were originally designed to target XIAP to modulate apoptosis; however, they also antagonize cIAP1 and other members of this protein family to varying degrees. cIAP1, an E3 ubiquitin ligase and member of the IAP family, regulates the activation of the non-canonical nuclear factor κB (ncNF-κB) pathway, driving expression of a specific set of genes that govern immune function.[24–27]

We previously reported that Smac mimetic compounds that can target the inhibitor of apoptosis protein cIAP1 (Birc2) harbor LRA activity.[28] Specifically, this previous study revealed that genetic or pharmacological antagonism of cIAP1 promoted ncNF-κB-dependent activation of the HIV-1 long terminal repeat (LTR), an activity that was also found to potently reactivate latent HIV-1. Based on this initial work, we now report the preclinical development and characterization of a bivalent next-generation Smac mimetic compound, Ciapavir (SBI-0953294), that was specifically optimized to enhance LRA activity and drug-like properties for *in vivo* reversal of the latent HIV-1 reservoir.

RESULTS

Bivalent Smac Mimetics Harbor Greater Potency as LRAs Than Monovalent Compounds

We have previously demonstrated that latency reversal of HIV-1 can be promoted in *in vitro* and *ex vivo* systems through pharmacological manipulation of the non-canonical NF-κB pathway using the Smac mimetic compound SBI-0637142.[28] This molecule modestly induced HIV-1 latency *ex vivo* in $CD4^+$ T cells from ART-suppressed aviremic HIV-infected patients as a single agent; however, robust activity was observed when administered in combination with the HDACi panobinostat. This suggested that SBI-0637142 likely possesses suboptimal efficacy to effectively mediate latency reversal as a single agent *in vivo*.

To develop novel compounds with optimized LRA efficacies, we first assessed the activity of previously developed Smac mimetics. Compounds in this class were originally designed to primarily target XIAP to relieve caspase inhibition and promote apoptosis, though certain newer molecules are designed to target both XIAP and cIAP1/2.[21,29,30] We compared SBI-0637142 with seven Smac mimetic molecules that have been developed as cancer therapeutics (Figure 1A), focusing on compounds that have entered phase I and/or phase II clinical trials targeting cancer, including GDC-0152, LCL-161, birinapant, AT-406, and ASTX660.[29,31–36] In addition, we included two compounds, BV6 and SM-164, that have shown efficacy as anticancer drugs in preclinical studies.[37–39] Smac mimetics that have been tested in clinical studies demonstrated acceptable safety profiles with some dose-limiting toxicities reported.[33,40–42]

Although exhibiting a wide range of activities in the latently infected Jurkat cell line 2D10,[43] none of the tested molecules exceeded the potency of SBI-0637142, with some clinically evaluated compounds showing little, if any, LRA activity (Figure 1B). Thus, the data indicate that Smac mimetic compounds that have been developed as anticancer agents may not necessarily be efficacious as LRAs. Interestingly, the structure-activity relationship (SAR) of the compounds clearly differentiated monovalent and bivalent molecules based on their observed potency and efficacy. Bivalent Smac mimetics have been proposed to bind to two available binding motifs in IAP proteins (BIR domains), either intra- or intermolecularly, thereby significantly enhancing the potency of these small molecules (see Discussion).[21,29,38,44] The monovalent compounds, LCL-161, GDC-0152, AT-406, and ASTX660, that bind only one BIR domain of IAP proteins exhibited notably lower LRA activity. In contrast, the bivalent compounds SM-164, birinapant, and BV-6 reached comparable levels of GFP-positive 2D10 cells at 10- to 100-fold lower concentrations (Figure 1B). None of the compounds caused detectable cytotoxicity under the conditions tested, with exception of BV-6 at 20 μM (Figure S1A). Critically, we observed that LRA activity of each of these molecules was commensurate with cIAP1 degradation (Figure 1C). Interestingly, SBI-0637142, a monovalent compound, continued to show equivalent or better potency than SM-164, the most potent bivalent compound in this evaluation. This observation led us to hypothesize that a bivalent structure based on SBI-0637142 could further improve the LRA activity of this compound.

Development of a Smac Mimetic Compound Optimized for HIV Latency Reversal Activity

We performed a detailed structural analysis to evaluate the binding mode of the monomeric inhibitor SBI-0637142 using *in silico* modeling based on the crystal structure of cIAP1 BIR3.[22] From these investigations, we concluded that a previously described linker in the P4 position[21] could be employed to create a dimeric version of SBI-0637142 with enhanced potency (Figure 1D). Based on this, we designed and synthesized a bivalent Smac mimetic, SBI-0953294, which we have termed Ciapavir (cIAP1 antagonist for viral reactivation; Figure 1D). A comparison of the first- and second-generation compounds in the 2D10 Jurkat latency model confirmed that the bivalent molecule Ciapavir exhibits substantially greater potency and efficacy as an LRA, inducing comparable levels of latency reversal at concentrations 10- to 1,000-fold lower than the first-generation molecule SBI-0637142 (Figure 1E), without an increase in cytotoxicity (Figure S1B). Ciapavir reached >65% of the LRA activity of phorbol 12-myristate 13-acetate (PMA)/ionomycin treatment (Figure S1C). Furthermore, genetic ablation of NF-κB-inducing kinase (NIK), a kinase essential for ncNF-κB activation, was sufficient to reverse Ciapavir LRA activity in this system (Figure 1F), indicating that this second-generation

(F) The LRA activity of SBI-0637142 and Ciapavir is dependent on the NIK signaling axis. 2D10 cells, unmodified (wt) and with a deletion of the NIK gene (ΔNIK), were treated with 1 μM SBI-0637142, 1 μM Ciapavir, or 10 nM bryostatin-1 for 48 h. Data represent mean and SD of three biological replicates. Statistical significance was analyzed by two-way ANOVA with Dunnett's multiple comparison test (n = 3).

(G) Ciapavir acts synergistically with JQ1 and I-BET151 to reverse HIV latency. 2D10 cells were treated with compound combinations for 48 h prior to analysis of cell viability and LRA activity (see Figure S1B). Synergy is shown as excess over Bliss (EOB). Values greater than 0 indicate positive synergy of the compounds. Gray fields indicate cell viability <70%. Heatmaps represent average values of two experiments.

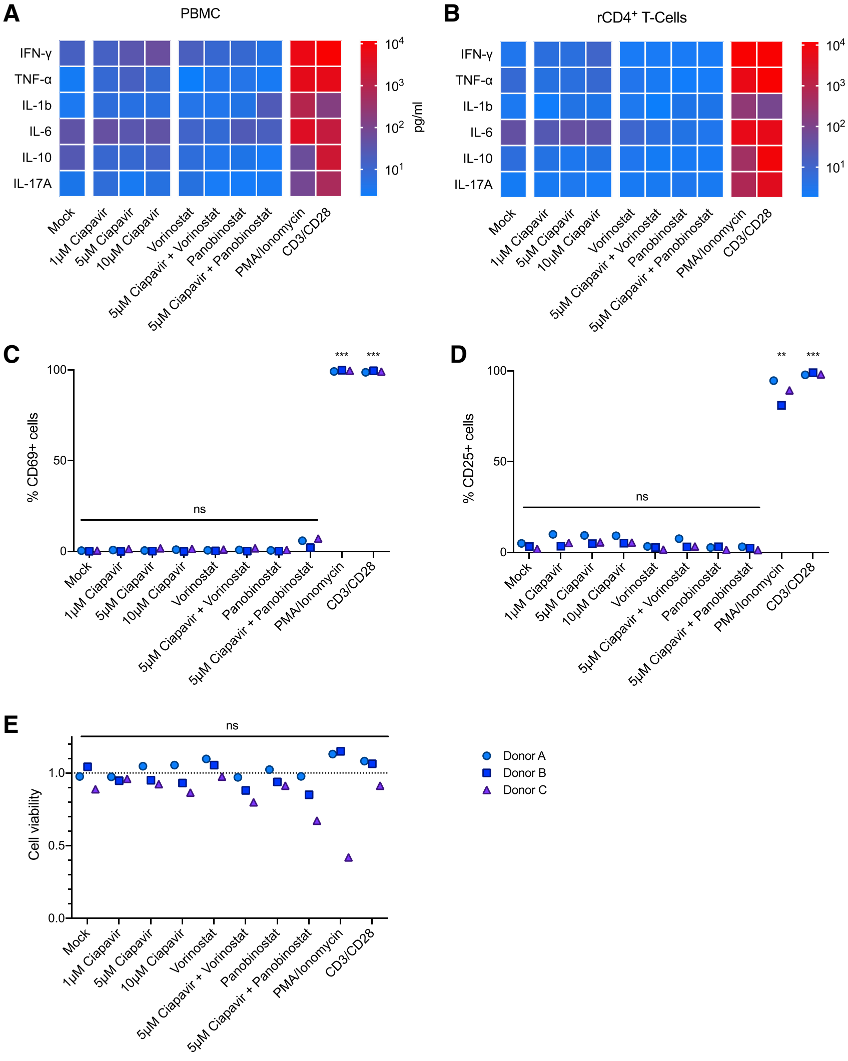

Figure 2. Ciapavir Does Not Induce Cytokine Release or T Cell Activation

Human PBMCs and rCD4$^+$ T cells from three healthy donors (n = 3) were treated with Ciapavir at the indicated concentrations, 500 nM vorinostat, 40 nM panobinostat, or combinations thereof, for 24 h. 50 ng/mL PMA and 1 μM ionomycin, or anti-CD3/CD28 antibody-coated beads, served as positive controls.

(A and B) Heatmaps represent mean cytokine levels measured in the culture supernatant of PBMCs (A) or rCD4$^+$ T cells (B) from tested donors (see Figure S2 for detailed results).

(C and D) rCD4$^+$ T cells treated as indicated were analyzed for CD69 (C) and CD25 (D) expression by flow cytometry.

(E) Viability of rCD4$^+$ T cells following treatment was assessed by measuring cellular ATP levels. Values were normalized to untreated cells from each donor. Significance was assessed with a one-way ANOVA using Dunnett's multiple comparison correction (n = 3).

molecule is mediating LRA activity through activation of the ncNF-κB pathway, consistent with the previously described mechanism reported by our group.[28]

Ciapavir Synergizes with Epigenetic Regulators to Enhance HIV-1 Latency Reversal

Similar to combinatorial ART, effective latency reversal as part of a curative therapy may ultimately require the combination of multiple LRAs to maximize efficacy.[45] We previously determined that the Smac mimetic SBI-0637142 synergizes with the HDACis vorinostat and panobinostat.[28] Here, we evaluated combinations of Ciapavir with two other well-established classes of LRAs, bromodomain and extraterminal domain inhibitors (BETi), and PKC agonists (PKCas) (Figures 1G and S1D). We observed potent Bliss synergy[46] of Ciapavir with the BETi JQ1 and I-BET151, with excess over Bliss (EOB) values greater than 0.6. Combinations of Ciapavir with the PKCas bryostatin-1 or ingenol-3-angelate, by contrast, exhibited concomitant toxicity that precluded efficient synergy.

Treatment with Ciapavir Does Not Trigger Cytokine Release or T Cell Activation

Although previous phase I and II clinical trials evaluating several Smac mimetics as cancer therapeutics generally determined these compounds to be safe for administration, cytokine release has been observed upon treatment with high doses of LCL-161.[33,34,42] Thus, we assessed the impact of Ciapavir treatment on cytokine levels in human peripheral blood mononuclear cells (PBMCs) and resting CD4$^+$ (rCD4$^+$) T cells. These data reveal that Ciapavir, alone or in combination with the HDACi vorinostat or panobinostat, does not induce significant cytokine release in PBMCs or rCD4$^+$ T cells at doses sufficient to trigger pathway activation (Figures 2A, 2B, and S2; data not shown). Importantly, the analysis of Ciapavir-treated rCD4$^+$ T cells by flow cytometry did not detect a significant increase in the expression of the early and late activation markers CD69 (Figure 2C) and CD25 (Figure 2D), supporting the conclusion that Ciapavir also does not mediate T cell activation *ex vivo*. This is of particular relevance because a number of compounds with reported LRA activity, notably PKC agonists, such as bryostatins or ingenols, have been shown to induce T cell activation,[47,48] representing a liability for their clinical application. Moreover, we find that treatment with Ciapavir did not negatively affect the viability of rCD4$^+$ T cells (Figure 2E).

In Vivo Evaluation Indicates Favorable Pharmacokinetic Properties of Ciapavir

To facilitate preclinical development of Ciapavir and evaluate the compound in *in vivo* efficacy models, we assessed the

CellPress
OPEN ACCESS

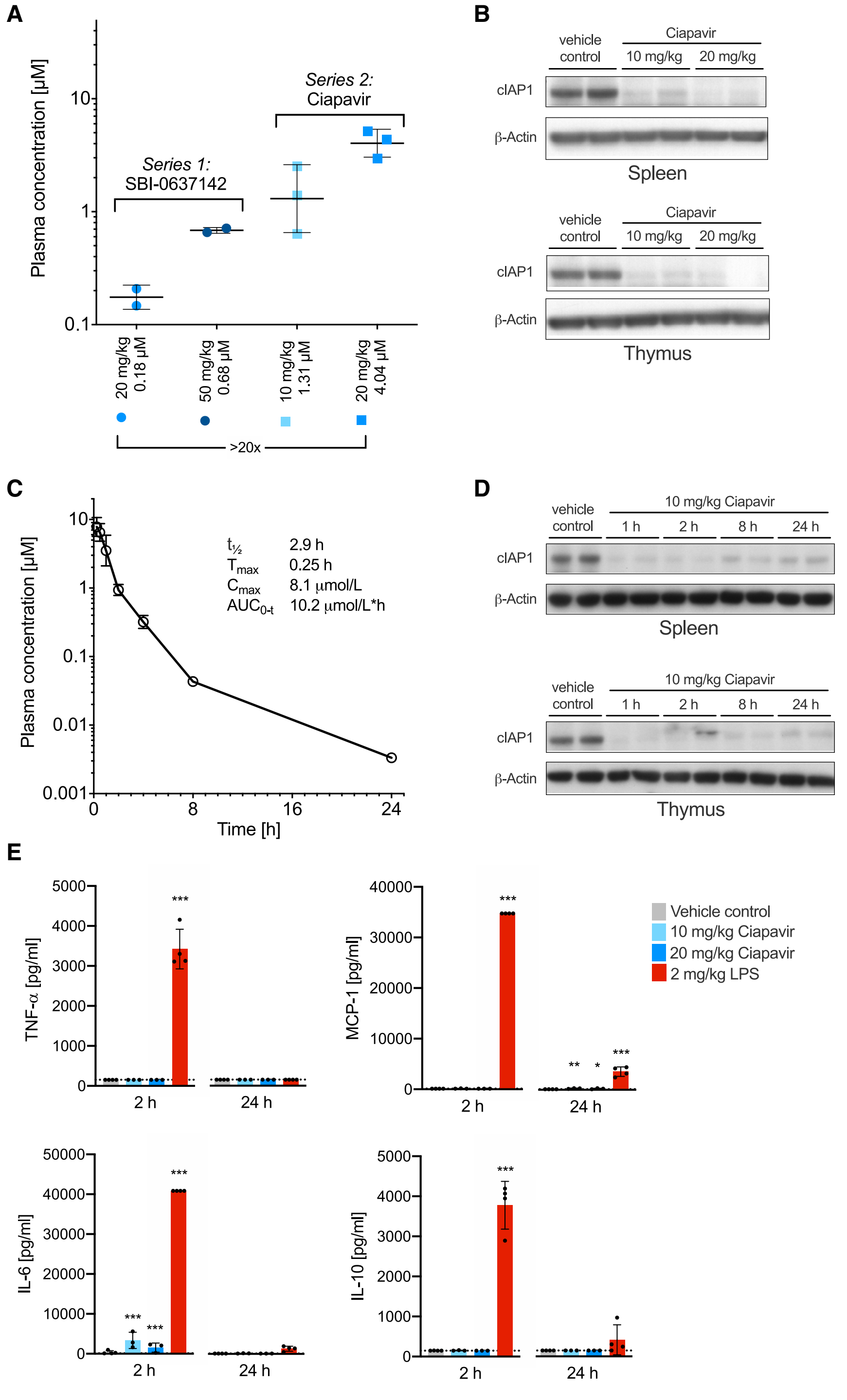

Figure 3. Ciapavir-Mediated Sustained Target Engagement *In Vivo* in Mice

(A) Plasma exposure of SBI-0637142 and Ciapavir after 10 and 20 mg/kg intraperitoneal dosage at 2 h. Error bars represent mean ± SD with n = 2 (SBI-0637142) and n = 3 (Ciapavir) in each group.

(B) Western blot showing cIAP1/2 degradation in spleen and thymus 2 h after treatment. Samples of two mice are shown for each condition.

(C) Pharmacokinetic time course of Ciapavir in mice after 10 mg/kg dosed intraperitoneally (n = 3; geometric mean ± geometric SD). Additional PK parameters are detailed in Table S1.

(D) Ciapavir treatment leads to sustained cIAP1/2 degradation over 24 h. cIAP1 levels in spleen and thymus of mice treated with 10 mg/kg Ciapavir by i.p. dosing for the indicated amount of time were analyzed by western blot. Samples of two mice are shown for each condition.

(E) C57BL/6 mice were dosed (i.p.) with 10 and 20 mg/kg Ciapavir. 2 mg/kg LPS served as positive control. Serum samples were isolated 2 and 24 h after compound administration and analyzed for cytokine levels. Graphs show mean and SD of four animals. Symbols represent values of individual animals. Detection limit is indicated by dotted line. Log transformed data were analyzed with a two-way ANOVA using Dunnett's multiple comparison correction (n = 4).

pharmacokinetic (PK) properties of the compound in mice. The PK studies revealed significantly greater *in vivo* plasma exposure in mice than the first-generation compound, SBI-0637142. Intraperitoneal (i.p.) injections of C57BL/6 mice with Ciapavir resulted in an approximately 20-fold increase in plasma concentrations after 2 h compared to the equivalent dose of SBI-0637142 (Figure 3A). Both doses of Ciapavir resulted in target engagement in mice 2 h after compound administration, as assessed by cIAP1 protein degradation (Figure 3B). Ciapavir at 10 mg/kg by i.p. dosing displayed a half-life ($t_{1/2}$) of 2.9 h (Figure 3C; Table S1) and enabled sustained cIAP1 degradation over at least 24 h (Figure 3D).

An assessment of cytokine levels indicated that Ciapavir treatment did not lead to substantially increased cytokine levels (Figure 3E) in mice, although LPS treatment resulted in robust cytokine release after 2 h. Taken together, these data indicate that systemic exposure of Ciapavir is sufficient to enable robust target engagement and, based on *in vitro* studies, latency reversal. Importantly, Ciapavir does not trigger cytokine release and is not associated with observable adverse events at the evaluated doses (data not shown). Therefore, we considered Ciapavir a suitable candidate for *in vivo* evaluation of LRA efficacy in a humanized mouse model of HIV-1 latency.

Latency Reversal in a Humanized Mouse Model of HIV Latency

BLT mice were constructed as previously described[10,49–51] and subsequently infected with an X4-tropic strain of HIV-1

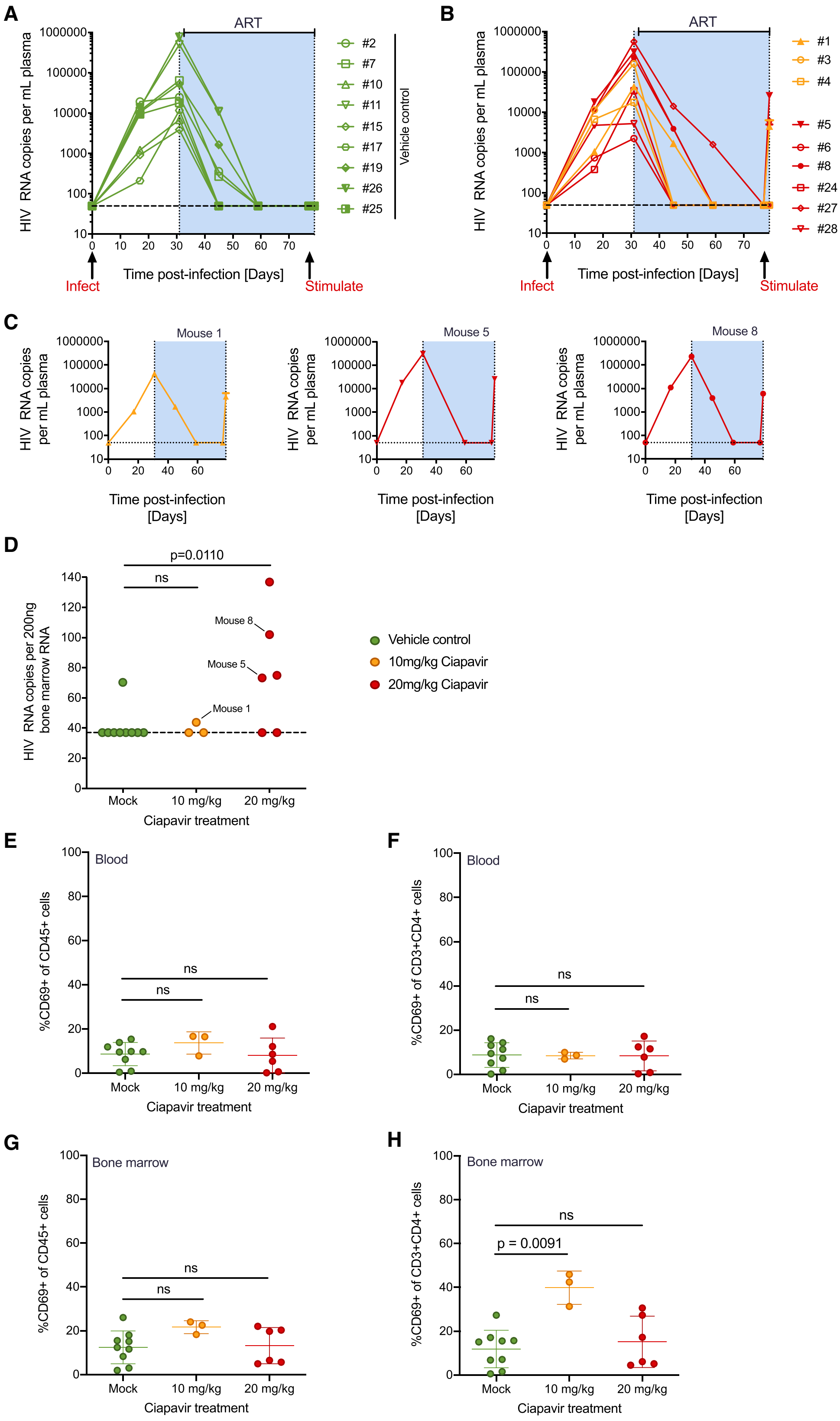

(legend on next page)

CellPress
OPEN ACCESS

based on NL4-3 that expresses a hemagglutinin tag in place of vpr.[52,53] Daily ART was initiated 4 weeks post-infection, and animals were observed to have suppressed viral loads following 7 weeks of treatment. ART was maintained to prevent viral spread, and either Ciapavir (3 mice at 10 mg/kg and 6 mice at 20 mg/kg) or vehicle control (9 mice) was administered to the animals by i.p. injection. At 2 days post-administration, mice were sacrificed and RNA from plasma and bone marrow samples was subjected to quantitative RT-PCR with HIV-1 gag-specific primers. Peripheral blood and bone marrow samples were also analyzed by flow cytometry for human immune cell composition and activation state using a panel of antibodies specific for the human cell surface markers: CD45; CD3; CD4; CD8; and CD69. Percentages of overall human $CD45^+$ immune cells, human T cells ($CD45^+CD3^+$), and human $CD4^+$ T cells ($CD45^+CD3^+CD4^+$) did not differ significantly between treatment groups (Figures S3A–S3C). Viral loads for all control (Figure 4A) and treated animals (Figure 4B) are shown. Three of the nine mice treated with Ciapavir (Figure 4C) exhibited increases in plasma RNA at the 48-h necropsy time point, although none of the other animals (including the nine vehicle control mice) had quantifiable plasma viremia. Evaluation of bone marrow RNA also showed a significant increase in HIV expression in four of the six mice in the 20 mg/kg Smac mimetic treatment group (Figure 4D). Despite the capacity of this compound to induce expression of latent HIV-1 *in vivo*, evidence for generalized immune activation in the animals treated with the Smac mimetic was minimal (Figures 4E–4H and S3D–S3H). The early activation marker CD69 was modestly upregulated in human cells present in the peripheral blood or bone marrow of animals treated with 10 mg/kg, but not significantly increased at the 20 mg/kg dose. We observed modest activation of a small set of cytokines analyzed (8/38), with eosinophil-activating interleukin-5 (IL-5) and anti-inflammatory IL-10 cytokines representing the most significant increases (Figure S3H). These data underscore that the latency reversal activity of Ciapavir is not accompanied by activation of T cells and a broad immune response *in vivo*. Together, these results indicate that Ciapavir is capable of increasing latent HIV-1 expression in ART-treated BLT mice *in vivo* and may therefore prove useful in "shock and kill" approaches to HIV-1 cure.

DISCUSSION

Using genetic screening approaches, we previously reported the discovery of ncNF-κB signaling as a critical mediator of HIV-1 latency reversal through transcriptional regulation of the HIV-1 LTR.[28] Here, we further demonstrate that an optimized small-molecule antagonist of cIAP1, Ciapavir, can potently promote HIV-1 latency reversal activity *in vivo*. These studies provide proof of concept that HIV-1 latency can be safely and effectively reversed through pharmacological manipulation of the ncNF-κB pathway. Ciapavir belongs to a class of molecules collectively referred to as Smac mimetics or IAP antagonists. Currently, eight human IAPs have been identified: XIAP; cIAP1; cIAP2; ILP2; BRUCE/Apollon; survivin; NAIP; and ML-IAP, and targeting a subset of these proteins has been demonstrated to promote apoptosis in cancer cells.[33,34] Therefore, there has been a concerted effort to harness this activity to develop anticancer compounds, and to date, seven Smac mimetics have entered clinical trials.

We evaluated a set of Smac mimetics that have been developed as cancer therapeutics, and have progressed to clinical trials, in a HIV-1 latency model. These studies showed that nearly all of the molecules[29,31,35–38] exhibit suboptimal LRA activity in comparison to SBI-0637142,[28] suggesting that anticancer activities of Smac mimetics may not correspond to LRA activity. Smac mimetics were originally optimized to target XIAP in cancer therapies,[21,55] though more recently, Smac mimetics have been developed to target both XIAP and cIAP1, as well as other IAPs. Importantly, in contrast to XIAP, cIAP1 controls ncNF-κB signaling, which regulates immune functions unrelated to cell death. We have previously observed that activation of ncNF-κB signaling is necessary for Smac mimetic latency reversal activity,[28] and here, we find that degradation of cIAP1 directly correlates with LRA activity (Figure 1C). However, we cannot rule out additional mechanisms that may contribute to these differences, including differences in XIAP engagement. Further studies will be needed to reveal what, if any, impact Smac mimetic selectivity across IAPs has upon LRA efficacy. Taken together, these data suggest that the optimization of Smac mimetics as LRAs requires preclinical strategies that are distinct from approaches used for the development of Smac mimetic anticancer compounds.

Interestingly, assessment of Smac mimetics indicated that bivalent compounds generally exhibit significantly greater LRA activity than monovalent molecules. A direct comparison of the second-generation bivalent compound Ciapavir with its corresponding monovalent counterpart illustrates the dramatic increase in activity that is mediated by this structural modification. Although the affinities of individual IAP binding motifs are generally not affected by the bivalency of the molecule, the ability of a dimer to simultaneously interact with two adjacent binding domains in an IAP protein may contribute to a more stable interaction and enhanced activity.[21] This configuration likely mimics the homodimeric structure of Smac in the cell that facilitates the dimerization necessary to induce the E3 ligase activity of cIAP1. Although it is currently unclear whether this interaction occurs intra- or intermolecularly, binding of bivalent Smac

Figure 4. Viral Loads and RNA Expression in Humanized Mice Treated with Ciapavir

Mice were infected with HIV-1, treated with ART to suppress viral loads, and then administered with Ciapavir or vehicle control.

(A and B) Viral loads of control (A) and Ciapavir-treated (B) BLT mice.

(C) 3 responding mice showing increases in viral loads 2 days after administration of Ciapavir.

(D) Bone marrow HIV RNA levels, with responding mice 1, 5, and 8 indicated.

(E–H) Mice described were subjected to flow cytometry analysis at necropsy to analyze immune activation. CD69 expression is shown as percentage gated of all blood $CD45^+$ (human immune) cells (E) and in $CD4^+$ T cells only (F). Bone marrow samples were analyzed for CD69 expression using the same parameters (G and H).

See Figure S3 for mean fluorescence intensities. Error bars represent mean and SD (n = 9 for mock, n = 3 for 10 mg/kg Ciapavir, and n = 6 for 20 mg/kg Ciapavir). Statistics represent two-sided Mann-Whitney test (ns, not significant; $p > 0.05$).

mimetics has been shown to enhance the E3 ubiquitin ligase activity of IAP proteins.[21,29,38,44] In addition, Sun and colleagues[56] reported that, besides connecting two IAP binding motifs at a specific distance, the linker can affect the hydrophobic properties of a bivalent Smac mimetic, thereby impacting cell permeability and dramatically increasing the cellular activity of the molecule. Thus, we hypothesize that the bivalency of Ciapavir may be an important factor contributing to the latency-reversing activity of this compound and should be considered in the design of novel molecules in this class to target HIV-1 latency.

Different classes of LRAs, including HDACi and the PKC agonist bryostatin-1, have been evaluated in clinical trials.[57,58] However, due to adverse effects or a lack of efficacy, no clear candidates for a therapeutic HIV-1 latency reversal have been identified to date. Elevated viral RNA expression has been observed in response to the HDACi vorinostat,[4,6] and increases in plasma viremia have been described after administration of romidepsin and panobinostat,[7,8] though the effects were generally modest. Importantly, reductions of the reservoir size have not been reported to date. The PKC agonist bryostatin-1 has been evaluated in a phase I clinical trial of aviremic HIV-1 infected patients, although the drug was tested at suboptimal doses due to concerns regarding its toxicity. At the doses evaluated, bryostatin-1 was well tolerated but failed to show any impact on PKC activity or HIV-1 latency reversal.[59] Other clinical studies have reported severe adverse effects of bryostatin-1 that may preclude tolerability at higher doses.[11] Thus, although most compounds that have progressed to clinical development appear to lack sufficient efficacy at safe doses, these LRAs may be suitable candidates for combinatorial treatment regimens. Synergies between different classes of LRAs have previously been reported and, similar to highly active antiretroviral cocktail therapies, combinations of multiple agents may ultimately be required to achieve a broad activation of the viral reservoir *in vivo*.[45,60–62] We previously identified potent synergies between Smac mimetics and the HDACis vorinostat and panobinostat.[28] Because a number of molecules in this class, including vorinostat, panobinostat, and belinostat, have already received US Food and Drug Administration (FDA) approval as therapeutics for cancer, HDACis are attractive candidates for combinatorial therapies that could rapidly progress to clinical development. Additionally, bromodomain and extraterminal domain inhibitors (BETi) are epigenetic regulators that affect HIV-1 replication by preventing positive transcription elongation factor b (P-TEFb) from interacting with BRD4, thereby allowing Tat to bind P-TEFb and mediating HIV-1 transcriptional elongation.[63,64] Although molecules in this class have been evaluated only preclinically for HIV-1 latency reversal,[65] different BETis are being investigated in clinical trials as cancer therapeutics.[66] Because we find that Smac mimetics can strongly synergize with a variety of epigenetic regulators, combinations of Smac mimetics with both HDACi and BETi represent potential opportunities to deliver increased latency reversal activity, while minimizing toxicities due to reduced doses of the individual drugs. Interestingly, due to concomitant toxicity, the combination of bryostatin-1 or ingenol-3-angelate with a Smac mimetic does not suggest a potential for drug synergy, indicating that combinatorial treatment with PKC agonists may not be beneficial.

Humanized mice have proven to be versatile tools in the study of HIV-1 latency and *in vivo* evaluation of latency reversing agents, including PKC agonists and HDACis.[10,47,67] Preclinical evaluation of the *in vivo* PK and pharmacodynamic characteristics of Ciapavir indicated favorable properties for evaluation of this LRA in an *in vivo* model of HIV-1. Critically, the results of the BLT efficacy study found that Ciapavir triggered robust viral gene expression in a significant number of animals (Figure 4D) without observed adverse effects or overt immune activation. In comparison to two other major classes of LRA that have previously been tested in these murine models, Smac mimetics promoted strong latency reversal observed across several animals and minimal upregulation of the early T cell activation marker CD69 (Figures 4E–4H). In contrast, LRA activity was absent in humanized BLT mice treated with the histone deacetylase inhibitor panobinostat,[67] and *in vivo* administration of the PKC agonist bryostatin resulted in significant induction of CD69 in T cells.[10] Although additional in-depth toxicity studies for Ciapavir are required, these data indicate that there exists a therapeutic window for this class of small molecules, either alone or in combination with other LRAs, to promote significant levels of HIV-1 latency reversal *in vivo*. Importantly, current data suggest that HIV-1 reactivation alone will likely not be sufficient to mediate clearance of the latent reservoir.[68] Further investigation of agents that can work in concert with a Smac mimetic-based LRA regimen to promote immune-mediated or cytopathic clearance of the viral reservoir, including CAR-T therapies or manipulation of immunomodulatory cytokines, will be critical for the development of a curative strategy for HIV-1.

A recent publication reported the LRA activity *in vivo* of AZD5582, a structurally unrelated Smac mimetic molecule that also shows potent target engagement of cIAP1.[69,70] Nixon and colleagues observed *in vivo* latency reversal upon Smac mimetic treatment in a humanized mouse model and in aviremic SIV-infected rhesus macaques. Importantly, no evidence of generalized T cell activation was observed in either study, further underscoring a favorable safety outlook for this class of LRAs. Taken together, these results provide *in vivo* proof of concept for the therapeutic targeting of ncNF-κB signaling to reverse HIV-1 latency. Furthermore, data presented in this study indicate that Ciapavir harbors sufficient drug-like, safety, and efficacy properties to advance to preclinical and clinical development as a HIV-1 latency reversing agent.

Limitations of Study

The first-generation Smac mimetic SBI-0637142 was previously found to harbor potent LRA activity when applied in combination with an HDACi in cells isolated from ART-suppressed, HIV-infected individuals. Although we find that Ciapavir, a more potent derivative of SBI-0637142, can effectively promote HIV-1 latency reversal *in vivo* as a monotherapy, further studies are required to determine whether Ciapavir harbors similar properties in patient-derived cells, both as a single agent and in combination with other LRAs, and whether these *ex vivo* endpoints correlate with *in vivo* efficacies.

In addition, although these data provide proof of concept for the LRA activity of Ciapavir *in vivo*, certain limitations of the experimental conditions used in this humanized mouse study

require consideration.[71] The low frequency of latently infected resting human CD4^{+} T cells precluded a comprehensive investigation of latency reversal in a broader range of tissues in individual animals. A study encompassing a significantly larger cohort of mice will enable the elucidation of the tissue-associated reservoir that can be targeted by Ciapavir *in vivo* by providing necessary statistical power. Finally, further studies characterizing the impact of Ciapavir on viral reservoir size and clearance will indicate whether implementation of parallel "kill" strategies, including immune-based therapies, will be required for the development of a successful therapeutic regimen to achieve a functional cure for HIV-1.

STAR★METHODS

Detailed methods are provided in the online version of this paper and include the following:

- KEY RESOURCES TABLE
- RESOURCE AVAILABILITY
  - Lead Contact
  - Materials Availability
  - Data and Code Availability
- EXPERIMENTAL MODEL AND SUBJECT DETAILS
  - Cells
  - Mice (pharmacokinetic and cytokine analysis)
  - Humanized Mice
- METHOD DETAILS
  - Chemical reagents
  - Latency reversal assays
  - Analysis of cIAP1 target engagement
  - Analysis of primary human cells
  - *In vivo* pharmacokinetics and cytokine analysis
  - Humanized mouse studies
  - Bone Marrow RT-dPCR
  - Flow cytometry (humanized mouse studies)
  - Plasma cytokine assay
- QUANTIFICATION AND STATISTICAL ANALYSIS

SUPPLEMENTAL INFORMATION

Supplemental Information can be found online at https://doi.org/10.1016/j.xcrm.2020.100037.

ACKNOWLEDGMENTS

We would like to thank Xiaomeng Wu, Hongying Chen, Paul D. DeJesus, and Andrey Rubanov for technical assistance and Douglas J. Sheffler for assistance with data analyses. L.P. received funding from the University of California San Diego Center for AIDS Research (CFAR), a National Institute of Health (NIH)-funded program (P30 AI036214), which is supported by the following NIH Institutes and Centers: NIAID; NCI; NIMH; NIDA; NICHD; NHLBI; NIA; NIGMS; and NIDDK. This study was supported by NIH/NIAID grant R01 AI124843 to V.P., N.D.P.C., J.A.Z., and S.K.C. Research reported in this publication was supported by National Cancer Institute of the NIH grant R01 CA195227 to N.D.P.C. and Cancer Center Support Grant P30 CA030199. Antiretroviral drugs for humanized mouse studies were kindly provided by Merck & Co. (raltegravir) and Gilead Sciences (TDF and FTC). This work was also supported by a generous grant from the James B. Pendleton Charitable Trust to support HIV/AIDS research. The content of this study is solely the responsibility of the authors and does not necessarily represent the official views of the NIH.

AUTHOR CONTRIBUTIONS

Conceptualization, L.P., N.D.P.C., J.A.Z., and S.K.C.; Investigation, L.P., M.D.M., P.T., A.J.P., D.H., J.T.K., M.S.A.S., M.D., C.C., and A.M.S.; Visualization, L.P., P.T., and M.D.M.; Supervision, V.P., N.D.P.C., J.A.Z., and S.K.C.; Funding Acquisition, L.P., V.P., N.D.P.C., J.A.Z., and S.K.C.; Writing – Original Draft, L.P. and S.K.C.; Writing – Review and Editing, L.P., M.D.M., P.T., D.H., A.M.S., V.P., N.D.P.C., J.A.Z., and S.K.C.

DECLARATION OF INTERESTS

The authors declare no competing interests. L.P., P.T., N.D.P.C., and S.K.C. are registered as inventors on patent US 10300074 B2, disclosing the use of inhibitor of apoptosis protein (IAP) antagonists in HIV therapy. N.D.P.C. is registered as inventor on patents US 9546174 B2, US 10047119 B2, and US 10544188 B2, disclosing inhibitor of apoptosis protein (IAP) antagonists. Additional patents are pending.

Received: February 5, 2020
Revised: April 1, 2020
Accepted: May 27, 2020
Published: June 23, 2020

REFERENCES

1. Margolis, D.M. (2010). Mechanisms of HIV latency: an emerging picture of complexity. Curr. HIV/AIDS Rep. *7*, 37–43.
2. Siliciano, J.D., and Siliciano, R.F. (2014). Recent developments in the search for a cure for HIV-1 infection: targeting the latent reservoir for HIV-1. J. Allergy Clin. Immunol. *134*, 12–19.
3. Spivak, A.M., and Planelles, V. (2018). Novel latency reversal agents for HIV-1 cure. Annu. Rev. Med. *69*, 421–436.
4. Archin, N.M., Liberty, A.L., Kashuba, A.D., Choudhary, S.K., Kuruc, J.D., Crooks, A.M., Parker, D.C., Anderson, E.M., Kearney, M.F., Strain, M.C., et al. (2012). Administration of vorinostat disrupts HIV-1 latency in patients on antiretroviral therapy. Nature *487*, 482–485.
5. Archin, N.M., Bateson, R., Tripathy, M.K., Crooks, A.M., Yang, K.H., Dahl, N.P., Kearney, M.F., Anderson, E.M., Coffin, J.M., Strain, M.C., et al. (2014). HIV-1 expression within resting CD4+ T cells after multiple doses of vorinostat. J. Infect. Dis. *210*, 728–735.
6. Elliott, J.H., Wightman, F., Solomon, A., Ghneim, K., Ahlers, J., Cameron, M.J., Smith, M.Z., Spelman, T., McMahon, J., Velayudham, P., et al. (2014). Activation of HIV transcription with short-course vorinostat in HIV-infected patients on suppressive antiretroviral therapy. PLoS Pathog. *10*, e1004473.
7. Søgaard, O.S., Graversen, M.E., Leth, S., Olesen, R., Brinkmann, C.R., Nissen, S.K., Kjaer, A.S., Schleimann, M.H., Denton, P.W., Hey-Cunningham, W.J., et al. (2015). The depsipeptide romidepsin reverses HIV-1 latency in vivo. PLoS Pathog. *11*, e1005142.
8. Rasmussen, T.A., Tolstrup, M., Brinkmann, C.R., Olesen, R., Erikstrup, C., Solomon, A., Winckelmann, A., Palmer, S., Dinarello, C., Buzon, M., et al. (2014). Panobinostat, a histone deacetylase inhibitor, for latent-virus reactivation in HIV-infected patients on suppressive antiretroviral therapy: a phase 1/2, single group, clinical trial. Lancet HIV *1*, e13–e21.
9. Bullen, C.K., Laird, G.M., Durand, C.M., Siliciano, J.D., and Siliciano, R.F. (2014). New ex vivo approaches distinguish effective and ineffective single agents for reversing HIV-1 latency in vivo. Nat. Med. *20*, 425–429.
10. Marsden, M.D., Loy, B.A., Wu, X., Ramirez, C.M., Schrier, A.J., Murray, D., Shimizu, A., Ryckbosch, S.M., Near, K.E., Chun, T.W., et al. (2017). In vivo activation of latent HIV with a synthetic bryostatin analog effects both latent cell "kick" and "kill" in strategy for virus eradication. PLoS Pathog. *13*, e1006575.
11. Morgan, R.J., Jr., Leong, L., Chow, W., Gandara, D., Frankel, P., Garcia, A., Lenz, H.J., and Doroshow, J.H. (2012). Phase II trial of bryostatin-1 in combination with cisplatin in patients with recurrent or persistent epithelial

ovarian cancer: a California cancer consortium study. Invest. New Drugs *30*, 723–728.

12. Borducchi, E.N., Cabral, C., Stephenson, K.E., Liu, J., Abbink, P., Ng'ang'a, D., Nkolola, J.P., Brinkman, A.L., Peter, L., Lee, B.C., et al. (2016). Ad26/MVA therapeutic vaccination with TLR7 stimulation in SIV-infected rhesus monkeys. Nature *540*, 284–287.
13. Borducchi, E.N., Liu, J., Nkolola, J.P., Cadena, A.M., Yu, W.H., Fischinger, S., Broge, T., Abbink, P., Mercado, N.B., Chandrashekar, A., et al. (2018). Antibody and TLR7 agonist delay viral rebound in SHIV-infected monkeys. Nature *563*, 360–364.
14. Lim, S.Y., Osuna, C.E., Hraber, P.T., Hesselgesser, J., Gerold, J.M., Barnes, T.L., Sanisetty, S., Seaman, M.S., Lewis, M.G., Geleziunas, R., et al. (2018). TLR7 agonists induce transient viremia and reduce the viral reservoir in SIV-infected rhesus macaques on antiretroviral therapy. Sci. Transl. Med. *10*, eaao4521.
15. Bekerman, E., Hesselgesser, J., Carr, B., Nagel, M., Hung, M., Wang, A., Stapleton, L., von Gegerfelt, A., Elyard, H.A., Lifson, J.D., and Geleziunas, R. (2019). PD-1 blockade and TLR7 activation lack therapeutic benefit in chronic simian immunodeficiency virus-infected macaques on antiretroviral therapy. Antimicrob. Agents Chemother. *63*, e01163-19.
16. Silke, J., and Meier, P. (2013). Inhibitor of apoptosis (IAP) proteins-modulators of cell death and inflammation. Cold Spring Harb. Perspect. Biol. *5*, a008730.
17. Finlay, D., Teriete, P., Vamos, M., Cosford, N.D.P., and Vuori, K. (2017). Inducing death in tumor cells: roles of the inhibitor of apoptosis proteins. F1000Res. *6*, 587.
18. Condon, S.M. (2011). Chapter 13 - The discovery and development of Smac mimetics-small-molecule antagonists of the inhibitor of apoptosis proteins. Annu. Rep. Med. Chem. *46*, 211–226.
19. Finlay, D., Vamos, M., González-López, M., Ardecky, R.J., Ganji, S.R., Yuan, H., Su, Y., Cooley, T.R., Hauser, C.T., Welsh, K., et al. (2014). Small-molecule IAP antagonists sensitize cancer cells to TRAIL-induced apoptosis: roles of XIAP and cIAPs. Mol. Cancer Ther. *13*, 5–15.
20. González-López, M., Welsh, K., Finlay, D., Ardecky, R.J., Ganji, S.R., Su, Y., Yuan, H., Teriete, P., Mace, P.D., Riedl, S.J., et al. (2011). Design, synthesis and evaluation of monovalent Smac mimetics that bind to the BIR2 domain of the anti-apoptotic protein XIAP. Bioorg. Med. Chem. Lett. *21*, 4332–4336.
21. Li, L., Thomas, R.M., Suzuki, H., De Brabander, J.K., Wang, X., and Harran, P.G. (2004). A small molecule Smac mimic potentiates TRAIL- and TNFalpha-mediated cell death. Science *305*, 1471–1474.
22. Vamos, M., Welsh, K., Finlay, D., Lee, P.S., Mace, P.D., Snipas, S.J., Gonzalez, M.L., Ganji, S.R., Ardecky, R.J., Riedl, S.J., et al. (2013). Expedient synthesis of highly potent antagonists of inhibitor of apoptosis proteins (IAPs) with unique selectivity for ML-IAP. ACS Chem. Biol. *8*, 725–732.
23. Welsh, K., Milutinovic, S., Ardecky, R.J., Gonzalez-Lopez, M., Ganji, S.R., Teriete, P., Finlay, D., Riedl, S., Matsuzawa, S., Pinilla, C., et al. (2016). Characterization of potent SMAC mimetics that sensitize cancer cells to TNF family-induced apoptosis. PLoS ONE *11*, e0161952.
24. Bonizzi, G., Bebien, M., Otero, D.C., Johnson-Vroom, K.E., Cao, Y., Vu, D., Jegga, A.G., Aronow, B.J., Ghosh, G., Rickert, R.C., and Karin, M. (2004). Activation of IKKalpha target genes depends on recognition of specific kappaB binding sites by RelB:p52 dimers. EMBO J. *23*, 4202–4210.
25. Mahoney, D.J., Cheung, H.H., Mrad, R.L., Plenchette, S., Simard, C., Enwere, E., Arora, V., Mak, T.W., Lacasse, E.C., Waring, J., and Korneluk, R.G. (2008). Both cIAP1 and cIAP2 regulate TNFalpha-mediated NF-kappaB activation. Proc. Natl. Acad. Sci. USA *105*, 11778–11783.
26. Sun, S.C. (2017). The non-canonical NF-κB pathway in immunity and inflammation. Nat. Rev. Immunol. *17*, 545–558.
27. Uno, M., Saitoh, Y., Mochida, K., Tsuruyama, E., Kiyono, T., Imoto, I., Inazawa, J., Yuasa, Y., Kubota, T., and Yamaoka, S. (2014). NF-κB inducing kinase, a central signaling component of the non-canonical pathway of NF-κB, contributes to ovarian cancer progression. PLoS ONE *9*, e88347.
28. Pache, L., Dutra, M.S., Spivak, A.M., Marlett, J.M., Murry, J.P., Hwang, Y., Maestre, A.M., Manganaro, L., Vamos, M., Teriete, P., et al. (2015). BIRC2/cIAP1 is a negative regulator of HIV-1 transcription and can be targeted by Smac mimetics to promote reversal of viral latency. Cell Host Microbe *18*, 345–353.
29. Condon, S.M., Mitsuuchi, Y., Deng, Y., LaPorte, M.G., Rippin, S.R., Haimowitz, T., Alexander, M.D., Kumar, P.T., Hendi, M.S., Lee, Y.H., et al. (2014). Birinapant, a smac-mimetic with improved tolerability for the treatment of solid tumors and hematological malignancies. J. Med. Chem. *57*, 3666–3677.
30. Oost, T.K., Sun, C., Armstrong, R.C., Al-Assaad, A.S., Betz, S.F., Deckwerth, T.L., Ding, H., Elmore, S.W., Meadows, R.P., Olejniczak, E.T., et al. (2004). Discovery of potent antagonists of the antiapoptotic protein XIAP for the treatment of cancer. J. Med. Chem. *47*, 4417–4426.
31. Cai, Q., Sun, H., Peng, Y., Lu, J., Nikolovska-Coleska, Z., McEachern, D., Liu, L., Qiu, S., Yang, C.Y., Miller, R., et al. (2011). A potent and orally active antagonist (SM-406/AT-406) of multiple inhibitor of apoptosis proteins (IAPs) in clinical development for cancer treatment. J. Med. Chem. *54*, 2714–2726.
32. Flygare, J.A., Beresini, M., Budha, N., Chan, H., Chan, I.T., Cheeti, S., Cohen, F., Deshayes, K., Doerner, K., Eckhardt, S.G., et al. (2012). Discovery of a potent small-molecule antagonist of inhibitor of apoptosis (IAP) proteins and clinical candidate for the treatment of cancer (GDC-0152). J. Med. Chem. *55*, 4101–4113.
33. Fulda, S. (2015). Promises and challenges of Smac mimetics as cancer therapeutics. Clin. Cancer Res. *21*, 5030–5036.
34. Fulda, S. (2017). Smac mimetics to therapeutically target IAP proteins in cancer. Int. Rev. Cell Mol. Biol. *330*, 157–169.
35. Ward, G.A., Lewis, E.J., Ahn, J.S., Johnson, C.N., Lyons, J.F., Martins, V., Munck, J.M., Rich, S.J., Smyth, T., Thompson, N.T., et al. (2018). ASTX660, a novel non-peptidomimetic antagonist of cIAP1/2 and XIAP, potently induces TNFα-dependent apoptosis in cancer cell lines and inhibits tumor growth. Mol. Cancer Ther. *17*, 1381–1391.
36. Weisberg, E., Ray, A., Barrett, R., Nelson, E., Christie, A.L., Porter, D., Straub, C., Zawel, L., Daley, J.F., Lazo-Kallanian, S., et al. (2010). Smac mimetics: implications for enhancement of targeted therapies in leukemia. Leukemia *24*, 2100–2109.
37. Li, W., Li, B., Giacalone, N.J., Torossian, A., Sun, Y., Niu, K., Lin-Tsai, O., and Lu, B. (2011). BV6, an IAP antagonist, activates apoptosis and enhances radiosensitization of non-small cell lung carcinoma in vitro. J. Thorac. Oncol. *6*, 1801–1809.
38. Lu, J., Bai, L., Sun, H., Nikolovska-Coleska, Z., McEachern, D., Qiu, S., Miller, R.S., Yi, H., Shangary, S., Sun, Y., et al. (2008). SM-164: a novel, bivalent Smac mimetic that induces apoptosis and tumor regression by concurrent removal of the blockade of cIAP-1/2 and XIAP. Cancer Res. *68*, 9384–9393.
39. Wu, M.S., Wang, G.F., Zhao, Z.Q., Liang, Y., Wang, H.B., Wu, M.Y., Min, P., Chen, L.Z., Feng, Q.S., Bei, J.X., et al. (2013). Smac mimetics in combination with TRAIL selectively target cancer stem cells in nasopharyngeal carcinoma. Mol. Cancer Ther. *12*, 1728–1737.
40. Amaravadi, R.K., Schilder, R.J., Martin, L.P., Levin, M., Graham, M.A., Weng, D.E., and Adjei, A.A. (2015). A phase I study of the SMAC-mimetic birinapant in adults with refractory solid tumors or lymphoma. Mol. Cancer Ther. *14*, 2569–2575.
41. DiPersio, J.F., Erba, H.P., Larson, R.A., Luger, S.M., Tallman, M.S., Brill, J.M., Vuagniaux, G., Rouits, E., Sorensen, J.M., and Zanna, C. (2015). Oral Debio1143 (AT406), an antagonist of inhibitor of apoptosis proteins, combined with daunorubicin and cytarabine in patients with poor-risk acute myeloid leukemia–results of a phase I dose-escalation study. Clin. Lymphoma Myeloma Leuk. *15*, 443–449.
42. Infante, J.R., Dees, E.C., Olszanski, A.J., Dhuria, S.V., Sen, S., Cameron, S., and Cohen, R.B. (2014). Phase I dose-escalation study of LCL161, an oral inhibitor of apoptosis proteins inhibitor, in patients with advanced solid tumors. J. Clin. Oncol. *32*, 3103–3110.

43. Pearson, R., Kim, Y.K., Hokello, J., Lassen, K., Friedman, J., Tyagi, M., and Karn, J. (2008). Epigenetic silencing of human immunodeficiency virus (HIV) transcription by formation of restrictive chromatin structures at the viral long terminal repeat drives the progressive entry of HIV into latency. J. Virol. *82*, 12291–12303.

44. Cossu, F., Milani, M., Mastrangelo, E., Vachette, P., Servida, F., Lecis, D., Canevari, G., Delia, D., Drago, C., Rizzo, V., et al. (2009). Structural basis for bivalent Smac-mimetics recognition in the IAP protein family. J. Mol. Biol. *392*, 630–644.

45. Laird, G.M., Bullen, C.K., Rosenbloom, D.I., Martin, A.R., Hill, A.L., Durand, C.M., Siliciano, J.D., and Siliciano, R.F. (2015). Ex vivo analysis identifies effective HIV-1 latency-reversing drug combinations. J. Clin. Invest. *125*, 1901–1912.

46. Bliss, C.I. (1939). The toxicity of poisons applied jointly. Ann. Appl. Biol. *26*, 585–615.

47. Marsden, M.D., and Zack, J.A. (2017). Humanized mouse models for human immunodeficiency virus infection. Annu. Rev. Virol. *4*, 393–412.

48. Spivak, A.M., Bosque, A., Balch, A.H., Smyth, D., Martins, L., and Planelles, V. (2015). Ex vivo bioactivity and HIV-1 latency reversal by ingenol dibenzoate and panobinostat in resting CD4(+) T cells from aviremic patients. Antimicrob. Agents Chemother. *59*, 5984–5991.

49. Lavender, K.J., Messer, R.J., Race, B., and Hasenkrug, K.J. (2014). Production of bone marrow, liver, thymus (BLT) humanized mice on the C57BL/6 Rag2(-/-)γc(-/-)CD47(-/-) background. J. Immunol. Methods *407*, 127–134.

50. Marsden, M.D., Kovochich, M., Suree, N., Shimizu, S., Mehta, R., Cortado, R., Bristol, G., An, D.S., and Zack, J.A. (2012). HIV latency in the humanized BLT mouse. J. Virol. *86*, 339–347.

51. Melkus, M.W., Estes, J.D., Padgett-Thomas, A., Gatlin, J., Denton, P.W., Othieno, F.A., Wege, A.K., Haase, A.T., and Garcia, J.V. (2006). Humanized mice mount specific adaptive and innate immune responses to EBV and TSST-1. Nat. Med. *12*, 1316–1322.

52. Ali, A., and Yang, O.O. (2006). A novel small reporter gene and HIV-1 fitness assay. J. Virol. Methods *133*, 41–47.

53. Jamieson, B.D., and Zack, J.A. (1998). In vivo pathogenesis of a human immunodeficiency virus type 1 reporter virus. J. Virol. *72*, 6520–6526.

55. Sun, H., Nikolovska-Coleska, Z., Yang, C.Y., Xu, L., Tomita, Y., Krajewski, K., Roller, P.P., and Wang, S. (2004). Structure-based design, synthesis, and evaluation of conformationally constrained mimetics of the second mitochondria-derived activator of caspase that target the X-linked inhibitor of apoptosis protein/caspase-9 interaction site. J. Med. Chem. *47*, 4147–4150.

56. Sun, H., Liu, L., Lu, J., Bai, L., Li, X., Nikolovska-Coleska, Z., McEachern, D., Yang, C.Y., Qiu, S., Yi, H., et al. (2011). Potent bivalent Smac mimetics: effect of the linker on binding to inhibitor of apoptosis proteins (IAPs) and anticancer activity. J. Med. Chem. *54*, 3306–3318.

57. Delagrèverie, H.M., Delaugerre, C., Lewin, S.R., Deeks, S.G., and Li, J.Z. (2016). Ongoing clinical trials of human immunodeficiency virus latency-reversing and immunomodulatory agents. Open Forum Infect. Dis. *3*, ofw189.

58. Spivak, A.M., and Planelles, V. (2016). HIV-1 eradication: early trials (and tribulations). Trends Mol. Med. *22*, 10–27.

59. Gutiérrez, C., Serrano-Villar, S., Madrid-Elena, N., Pérez-Elías, M.J., Martín, M.E., Barbas, C., Ruipérez, J., Muñoz, E., Muñoz-Fernández, M.A., Castor, T., and Moreno, S. (2016). Bryostatin-1 for latent virus reactivation in HIV-infected patients on antiretroviral therapy. AIDS *30*, 1385–1392.

60. Burnett, J.C., Lim, K.I., Calafi, A., Rossi, J.J., Schaffer, D.V., and Arkin, A.P. (2010). Combinatorial latency reactivation for HIV-1 subtypes and variants. J. Virol. *84*, 5958–5974.

61. Darcis, G., Kula, A., Bouchat, S., Fujinaga, K., Corazza, F., Ait-Ammar, A., Delacourt, N., Melard, A., Kabeya, K., Vanhulle, C., et al. (2015). An in-depth comparison of latency-reversing agent combinations in various in vitro and ex vivo HIV-1 latency models identified bryostatin-1+JQ1 and ingenol-B+JQ1 to potently reactivate viral gene expression. PLoS Pathog. *11*, e1005063.

62. Xing, S., and Siliciano, R.F. (2013). Targeting HIV latency: pharmacologic strategies toward eradication. Drug Discov. Today *18*, 541–551.

63. Boehm, D., Conrad, R.J., and Ott, M. (2013). Bromodomain proteins in HIV infection. Viruses *5*, 1571–1586.

64. Zhu, J., Gaiha, G.D., John, S.P., Pertel, T., Chin, C.R., Gao, G., Qu, H., Walker, B.D., Elledge, S.J., and Brass, A.L. (2012). Reactivation of latent HIV-1 by inhibition of BRD4. Cell Rep. *2*, 807–816.

65. Li, G., Zhang, Z., Reszka-Blanco, N., Li, F., Chi, L., Ma, J., Jeffrey, J., Cheng, L., and Su, L. (2019). Specific activation *in vivo* of HIV-1 by a bromodomain inhibitor from monocytic cells in humanized mice under antiretroviral therapy. J. Virol. *93*, e00233-19.

66. Alqahtani, A., Choucair, K., Ashraf, M., Hammouda, D.M., Alloghbi, A., Khan, T., Senzer, N., and Nemunaitis, J. (2019). Bromodomain and extra-terminal motif inhibitors: a review of preclinical and clinical advances in cancer therapy. Future Sci. OA *5*, FSO372.

67. Tsai, P., Wu, G., Baker, C.E., Thayer, W.O., Spagnuolo, R.A., Sanchez, R., Barrett, S., Howell, B., Margolis, D., Hazuda, D.J., et al. (2016). In vivo analysis of the effect of panobinostat on cell-associated HIV RNA and DNA levels and latent HIV infection. Retrovirology *13*, 36.

68. Kim, Y., Anderson, J.L., and Lewin, S.R. (2018). Getting the “kill” into “shock and kill”: strategies to eliminate latent HIV. Cell Host Microbe *23*, 14–26.

69. Hennessy, E.J., Adam, A., Aquila, B.M., Castriotta, L.M., Cook, D., Hattersley, M., Hird, A.W., Huntington, C., Kamhi, V.M., Laing, N.M., et al. (2013). Discovery of a novel class of dimeric Smac mimetics as potent IAP antagonists resulting in a clinical candidate for the treatment of cancer (AZD5582). J. Med. Chem. *56*, 9897–9919.

70. Nixon, C.C., Mavigner, M., Sampey, G.C., Brooks, A.D., Spagnuolo, R.A., Irlbeck, D.M., Mattingly, C., Ho, P.T., Schoof, N., Cammon, C.G., et al. (2020). Systemic HIV and SIV latency reversal via non-canonical NF-κB signalling in vivo. Nature *578*, 160–165.

71. Marsden, M.D. (2020). Benefits and limitations of humanized mice in HIV persistence studies. Retrovirology *17*, 7.

72. Zhang, Y., Huo, M., Zhou, J., and Xie, S. (2010). PKSolver: an add-in program for pharmacokinetic and pharmacodynamic data analysis in Microsoft Excel. Comput. Methods Programs Biomed. *99*, 306–314.

73. Bristol, G.C., Gao, L.Y., and Zack, J.A. (1997). Preparation and maintenance of SCID-hu mice for HIV research. Methods *12*, 343–347.

74. McCune, J.M., Namikawa, R., Kaneshima, H., Shultz, L.D., Lieberman, M., and Weissman, I.L. (1988). The SCID-hu mouse: murine model for the analysis of human hematolymphoid differentiation and function. Science *241*, 1632–1639.

75. Shimizu, S., Ringpis, G.E., Marsden, M.D., Cortado, R.V., Wilhalme, H.M., Elashoff, D., Zack, J.A., Chen, I.S., and An, D.S. (2015). RNAi-mediated CCR5 knockdown provides HIV-1 resistance to memory T cells in humanized BLT mice. Mol. Ther. Nucleic Acids *4*, e227.

76. Goswami, C.P., Cheng, L., Alexander, P.S., Singal, A., and Li, L. (2015). A new drug combinatory effect prediction algorithm on the cancer cell based on gene expression and dose-response curve. CPT Pharmacometrics Syst. Pharmacol. *4*, e9.

STAR★METHODS

KEY RESOURCES TABLE

REAGENT or RESOURCE	SOURCE	IDENTIFIER
Antibodies		
Anti-cIAP1	R&D Systems	Cat# AF8181; RRID: AB_2259001
Anti-GAPDH	R&D Systems	Cat# AF5718; RRID: AB_2278695
Anti-cIAP	R&D Systems	Cat# MAB3400; RRID: AB_2063803
PE anti-CD69	BioLegend	Cat# 310906; RRID: AB_314841
APC anti-CD25	BioLegend	Cat# 302610; RRID: AB_314280
Pacific Blue anti-human CD45 Antibody (HI30)	BioLegend	Cat# 304029; RRID: AB_2174123
APC/Cy7 anti-human CD3 Antibody (HIT3A)	BioLegend	Cat# 300318; RRID: AB_314054
PE anti-human CD4 Antibody (OKT4)	BioLegend	Cat# 317410; RRID: AB_571955
FITC anti-human CD69 Antibody (FN50)	BioLegend	Cat# 310904; RRID: AB_314839
PerCP/Cy5.5 anti-human CD8a Antibody (RPA-T8)	BioLegend	Cat# 301032; RRID: AB_893422
Dynabeads Human T-Activator CD3/CD28	ThermoFisher	Cat# 11132D
Bacterial and Virus Strains		
NL4-3 based HIV-1 modified to include HA instead of vpr	this study	n/a
Chemicals, Peptides, and Recombinant Proteins		
SBI-0637142	Vamos et al.[22]	n/a
Ciapavir (SBI-0953294)	this study	n/a
LCL-161	APExBio	Cat# A3541
AT-406	APExBio	Cat# A3019
SM-164	BioVision	Cat# B1816
BV-6	Selleck Chemicals	Cat# S7597
ASTX660	MedChemExpress	Cat# HY-109565
GDC-0152	Cayman Chemical	Cat# 17810-1
Vorinostat	BioGems	Cat# 1497894
Panobinostat	LC Laboratories	Cat# P-3703
JQ1	APExBio	Cat# A1910
I-BET151	BioVision	Cat# 2220
Ingenol-3-angelate	AdipoGen Life Sciences	Cat# AG-CN2-0012
Bryostatin-1	MilliporeSigma	Cat# 203811
Phorbol 12-myristate 13-acetate (PMA)	Sigma-Aldrich	Cat# P1585
Ionomycin	Sigma-Aldrich	Cat# I9657
Emtricitibine (FTC)	Gilead Sciences	n/a
Tenofovir disoproxil fumarate (TDF)	Gilead Sciences	n/a
Raltegravir	Merck	n/a
Critical Commercial Assays		
CellTiter-Glo Cell Viability Assay	Promega	Cat# G7573
LEGENDplex Mouse Inflammation Panel	BioLegend	Cat# 740150
Cytokine/Chemokine Magnetic Bead Panel - Premixed 38 Plex - Immunology Multiplex Assay	Millipore	Cat# HCYTMAG-60K-PX3838
QuantStudio 3D Digital PCR Master Mix v2	ThermoFisher	A26358
QuantStudio 3D Digital PCR 20K Chip Kit v2	ThermoFisher	A26316
Experimental Models: Cell Lines		
2D10	Pearson et al.[43]	n/a
2D10 ΔNIK	Pache et al.[28]	n/a

(Continued on next page)

Continued

REAGENT or RESOURCE	SOURCE	IDENTIFIER
Experimental Models: Organisms/Strains		
C57BL/6J mice	Jackson Laboratory	Stock# 000664
Triple KO (TKO) mice: B6.129S-Rag2^{tm1Fwa} Cd47^{tm1Fpl} Il2^{rgtm1Wjl}/J	Jackson Laboratory	Stock# 025730
Oligonucleotides		
5′-CCTTTTAGAGACATCAGAAGGCTGTAGACAAAT ACTGGG-3′	ThermoFisher	Gag-FAM
5′-GGGAGCTAGAACGATTCGCAGTTAAT-3′	ThermoFisher	Gag-F1
5′-ATAATGATCTAAGTTCTTCTGATCCTGTCTGAA GGGA-3′	ThermoFisher	Gag-R1
Software and Algorithms		
Prism 8	GraphPad	n/a
Attune NxT software	ThermoFisher	n/a
PK-solver for Excel	Zhang et al.[72]	n/a
QuantStudio 3D AnalysisSuite Cloud v3.0 Software	ThermoFisher	n/a
FlowJo (v10) Software	FlowJo, LLC	n/a
MILLIPLEX Analyst 5.1 Software	Millipore	n/a
Other		
EasySep Human Resting CD4$^+$ T Cell Isolation Kit	StemCell Technologies	Cat# 17962

RESOURCE AVAILABILITY

Lead Contact

Further information and requests for resources and reagents should be directed to and will be fulfilled by the Lead Contact, Sumit K. Chanda (schanda@SBPdiscovery.org).

Materials Availability

Materials generated in this study can be requested through the lead contact. Any transfer will be subject to a material transfer agreement (MTA) that will include reimbursement for applicable costs.

Data and Code Availability

This study did not generate datasets or code.

EXPERIMENTAL MODEL AND SUBJECT DETAILS

Cells

The latently infected Jurkat cell line 2D10[43] was obtained from Dr. Jonathan Karn (Case Western Reserve University). The generation of cells with a knockout of the *NIK* gene (2D10 ΔNIK) is described in Pache et al.[28] Peripheral blood mononuclear cells (PBMCs) were isolated by Ficoll density gradient centrifugation (Histopaque, Sigma Aldrich) from buffy coats of healthy human donors (San Diego Blood Bank). Resting CD4$^+$ T cells were isolated using the EasySep Human Resting CD4$^+$ T Cell Isolation Kit (Cat# 17962, StemCell Technologies). Cells were cultured at 37°C and 5% CO_2 in RPMI1640 supplemented with 10% FBS, 100 IU penicillin, 100 μg/mL streptomycin, 0.01 M HEPES, and 2 mM L-glutamine.

Mice (pharmacokinetic and cytokine analysis)

Adult female C57BL/6J were purchased from the The Jackson Laboratory and housed with free access to food and water on a 12 h light/dark cycle. All animal procedures were approved by the Sanford Burnham Prebys Medical Discovery Institute Institutional Animal Care and Use Committee and were performed according to the NIH guidelines for the Care and Use of Laboratory Animals.

Humanized Mice

Humanized mice experiments were approved by the University of California, Los Angeles Chancellor's Animal Research Committee (ARC). All experiments conformed to local and national regulatory standards. Humanized triple knockout bone marrow liver thymus (TKO-BLT) mice[49,53] were constructed by the UCLA humanized mouse core using techniques described previously[10,49,50,73,74]. In brief, B6.129S-*Rag2*^{tm1Fwa} *Cd47*^{tm1Fpl} *Il2rg*^{tm1Wjl}/J (TKO) mice, purchased from The Jackson Laboratory and bred at UCLA, were

irradiated with 500 rads and then transplanted under the kidney capsule with pieces of fetal thymus and liver tissue. Mice were then infused intravenously by retro-orbital injection with $5x10^5$ human fetal liver-derived $CD34^+$ cells isolated by immunomagnetic separation as previously described[10,75]. At this time, the mice were also infused intravenously by retro-orbital injection with $2x10^6$ splenocytes and $5x10^5$ bone marrow cells from a donor TKO mouse. If mice showed signs of anemia, they were transfused with additional splenocytes at 3- and 6-days post-surgery. At 8 weeks post-transplantation and approximately every 2 weeks thereafter mice were evaluated for reconstitution with human cells. Mice were bled as previously described[10,50] and peripheral blood mononuclear cells analyzed by flow cytometry. Mice were maintained in HEPA-filtered mouse racks in groups of up to 5 animals per cage. Male and female mice were included in the study to avoid systematic bias associated with sex as a biological variable. Mice were 3 months of age at time of initial transplant. Only mice that retained humanization throughout the experimental time course were included in the analysis.

METHOD DETAILS

Chemical reagents

SBI-0637142 was synthesized as previously described[22]. The synthesis of Ciapavir is described by D.H., Nicole Bata, Darren Finlay, Nicole Klinker, Allison S. Limpert, P.T., Luke Vickrey, Lester J. Lambert, Douglas J. Sheffler, Carol Burian, James Mason, Andrew D. Mesecar, Kristiina Vuori, and N.D.P.C. (unpublished data). LCL-161 and AT-406 were obtained from ApexBio, Birinapant and SM-164 from BioVision, BV-6 from Selleck Chemicals, ASTX660 from MedChemExpress, and GDC-0152 from Cayman Chemical. Vorinostat (suberanilohydroxamic acid, SAHA) and panobinostat were purchased from BioGems and LC Laboratories, respectively. JQ1 was purchased from Apexbio Technology, I-BET151 from BioVision, Ingenol-3-angelate from AdipoGen Life Sciences, and Bryostatin-1 from MilliporeSigma. All compounds were dissolved in dimethyl sulfoxide (DMSO, Fisher Scientific). Equal concentrations of DMSO were used as negative control.

Latency reversal assays

Compounds for dose response assays, adjusted for equal DMSO concentrations, were spotted in 384-well plates with a Labcyte Echo 555 Liquid Handler and $4x10^5$ 2D10 cells suspended in 50 μL RPMI were added to each well. After 48 h, GFP expression was analyzed with an Attune NxT flow cytometer and the Attune NxT software (ThermoFisher). Cell viability was assessed by analyzing forward and side scatter characteristics of the cells using flow cytometry, and by measuring cellular ATP levels. ATP levels were determined by adding CellTiter-Glo Cell Viability Assay reagent (Promega) to the cells and measuring luminescence using an EnSpire plate reader (PerkinElmer). Cell viability measurements were normalized to the average value of control samples mock-treated with DMSO.

Synergy of drug combinations was assessed using the Bliss independence model[46], that predicts that if two drugs D_A and D_B with experimentally determined fractional effects f_A and f_B have an additive effect, their expected fractional combinatorial effect is: $f_{AB} = f_A + f_B - (f_A \times f_B)$. Excess over Bliss (EOB) is calculated as the difference between the observed fractional effect of drugs D_A and D_B in combination $f^{obs}{}_{AB}$ and f_{AB} with $EOB = f^{obs}{}_{AB} - f_{AB}$. EOB values of ~0 indicate additive behavior, while values > 0 indicate synergistic behavior[76].

Analysis of cIAP1 target engagement

To measure cIAP1 protein degradation, 2D10 cells were treated with the indicated compound dilutions for 24 h. Cells were then lysed in radioimmunoprecipitation assay (RIPA) buffer and protein concentrations were determined using the Pierce BCA Protein Assay Kit (Life Technologies). Western blot analysis was performed on a Peggy Sue Automated Western Blot System (ProteinSimple) with sample concentrations adjusted to 1.8 μg/μL and using primary antibodies against cIAP1 (AF8181, R&D Systems) at a concentration of 5 μg/mL, and against GAPDH (AF5718, R&D Systems) at a concentration of 0.04 μg/mL as loading control.

Analysis of primary human cells

PBMC or resting CD4+ T cells were treated with DMSO, Ciapavir, 500 nM vorinostat, 40 nM panobinostat, or combinations thereof, or 50 ng/ml phorbol 12-myristate 13-acetate (PMA) and 1 μM ionomycin, or CD3/CD28 antibody-coated magnetic beads as positive controls, for 24 h. Cytokine levels were analyzed using the LEGENDplex Human Inflammation Panel 1 (BioLegend) and an Attune NxT flow cytometer (ThermoFisher). To assess the activation state of the $CD4^+$ T cells, cells were stained with a PE-labeled anti-CD69 antibody (Cat# 310906, BioLegend) and an APC-labeled anti-CD25 antibody (Cat# 302610, BioLegend), and analyzed by flow cytometry using an Attune NxT flow cytometer and the Attune NxT software (ThermoFisher). Viability of cells was measured using CellTiter-Glo Cell Viability Assay reagent (Promega) and an EnSpire plate reader (PerkinElmer).

In vivo pharmacokinetics and cytokine analysis

Compounds were formulated in 0.9% sterile sodium chloride (Hospira, Lake Forest, IL) and injected intraperitoneally (i.p.) into adult female C57BL/6J mice at doses of 10 or 20 mg/kg. Blood samples were collected retro-orbitally at indicated time points and plasma was separated by centrifugation. Livers, thymus, and spleen were collected postmortem. Plasma samples were extracted with acetonitrile:water 4:1 with 0.1% formic acid containing indomethacin as an internal standard. Samples were centrifuged and

supernatants were diluted with acetonitrile:water and analyzed via LC-MS/MS on a Shimadzu Nexera X2 HPLC coupled to an AB Sciex 6500 QTRAP. The results were analyzed with PK-solver for Excel[72].

Western blot analysis of mouse tissue was performed as follows. Tissue was suspended in RIPA buffer supplemented with protease inhibitor cocktail (P8340, MilliporeSigma) and 0.2 mM PMSF and homogenized in a PowerGen 125 Homogenizer (Fisher Scientific). Samples were sonicated and centrifuged for 20 min at 4°C and 14,000 xg. Supernatants were collected, and protein concentrations were determined using the Pierce BCA Protein Assay Kit (Life Technologies). SDS-PAGE and western blot analysis was conducted following standard protocols using a cIAP-specific antibody (MAB3400, R&D Systems). To analyze cytokine induction, adult C57BL/6J mice were dosed with vehicle control, 10 mg/kg or 20 mg/kg Ciapavir, or 2 mg/kg LPS (L4391, E.coli serotype 0111:B4, Sigma-Aldrich). Blood samples were collected retro-orbitally at indicated time points and plasma was separated by centrifugation. Cytokine levels were determined using the LEGENDplex Mouse Inflammation Panel (BioLegend).

Humanized mouse studies

Humanized mice were infected with an X4-tropic strain of HIV-1 based on NL4-3 modified to include HSA in place of vpr[53], then further modified to include HA in place of HSA[52] with random 21 nucleotide sequence inserted in non-coding region beside HSA gene (M.D. Marsden, et al., 2018, Strat. HIV Cure, abstract). HIV RNA copy numbers in plasma at each bleed were quantified using RT-PCR performed by the UCLA AIDS Institute virology core as previously described[10]. After 4 weeks of infection, mice were treated with ART in animal feed consisting of emtricitibine (FTC) at 0.5 mg/g of feed, tenofovir disoproxil fumarate (TDF) at 0.75 mg/g of feed, and raltegravir at 1 mg/g of feed for a further approximately 7 weeks. Mice were randomized based on pre-ART viral load, to ensure similar viral loads in each treatment group. Smac mimetic compound was introduced between 76 and 78 days post-infection by intraperitoneal injection and mice were sacrificed 48 h later for tissue processing. Necropsies (2 days after compound administration) were staggered, and performed at day 78, 79, or 80 post-infection. At this point mice were anesthetized with isoflurane and then exsanguinated by intracardiac bleed using a 1 mL syringe and 25G ½ inch needle rinsed with 0.5 M EDTA. Resultant blood was transferred into a 1.5 mL screw-capped tube containing 2 µL of 0.5 M EDTA. Animals were then euthanized, and bones removed by dissection. For bone marrow cell extraction, the femur and tibia were cut at both ends with sharp sterile scissors. Cells were collected by centrifugation of the bones at 9168 xg for 15 s into a collection tube. Bone marrow cell pellet was resuspended in 5 mL of RF10 media consisting of RPMI media (Invitrogen) containing 10% fetal bovine serum (Omega Scientific), 100 U/mL of penicillin, and 100 µg/mL of streptomycin (pen/strep: Invitrogen) then filtered through a 40 µm filter. Cells were then washed, and red blood cells lysed by resuspending pellet in 2 mL of Ammonium Chloride Solution lysis buffer (StemCell Technologies). The cells were then briefly vortexed and incubated at room temperature for 5 min, before pelleting by centrifugation at 1300 xg. Blood was centrifuged at 1300 xg and the upper plasma layer was collected, aliquoted and stored at −80°C. The central 150 µL layer containing white blood cells was transferred into a new 1.5 mL screw-capped tube and then 1 mL of Ammonium Chloride Solution lysis buffer was added to each tube. Tubes were then briefly vortexed and incubated at room temperature for 5 min, before pelleting by centrifugation at 1300 xg. Blood and bone marrow cells were subjected to a final wash with RF10 media and either used for flow cytometry as described below or suspended in RLT buffer (QIAGEN) for RNA storage and then frozen at −80°C.

Bone Marrow RT-dPCR

HIV RNA copy numbers in bone marrow were quantified using chip-based reverse transcription digital PCR (RT-dPCR) and HIV-1 gag-specific primers. Cell-associated RNA (CA-RNA) was extracted from bone marrow cells using QIAGEN RNeasy Mini Kit according to manufacturer's protocol. RT-dPCR reaction mixture was loaded into QuantStudio 3D Digital PCR Chip and run on the QuantStudio 3D Digital PCR System following manufacturer's instructions (ThermoFisher). RT-dPCR reaction was performed in 20 µL containing 10 µL 2x QuantStudio 3D Mastermix (ThermoFisher), 2 µL Superscript VILO (ThermoFisher), 1 µL Taqman 20x Mastermix containing 900 nM primers and 250 nM probe (Thermofisher), and 200 ng of template RNA with the following cycling conditions: 30 min at 50°C, 10 min at 96°C, 40 cycles each consisting of a 30 sec at 96°C followed by 60°C for 2 min, a final 2 min extension at 60°C, and final hold at 10°C. Cycling chips were analyzed immediately or stored at 4°C overnight until analysis. Raw fluorescence data for each well was exported and analyzed using the manufacturer's software (QuantStudio 3D AnalysisSuite Cloud Software). A no-template negative control and a positive control containing plasmid DNA were used to set the negative and positive thresholds, respectively. The number of template copies per unit volume was estimated from the number of positive events detected in the corresponding chip and the number of total accepted partitions.

Flow cytometry (humanized mouse studies)

Samples of $2x10^5$ cells were suspended in 50 µL of a 1:1 dilution of phosphate buffered saline (PBS):Human AB serum (Sigma). The following fluorescent conjugated antibodies were then added: Pacific Blue anti-human CD45 Antibody (HI30, Biolegend); APC/Cy7 anti-human CD3 Antibody (HIT3A- Biolegend); PE anti-human CD4 Antibody (OKT4, Biolegend); PerCP/Cy5.5 anti-human CD8a Antibody (RPA-T8- Biolegend); FITC anti-human CD69 Antibody (FN50, Biolegend). Cells were then incubated at 4 °C for 20 min, washed with PBS, and resuspended in 2% paraformaldehyde. Stained samples were stored at 4 °C until analysis was performed using an Attune NxT (ThermoFisher) flow cytometer. Data was analyzed using FlowJo (v10) software.

Plasma cytokine assay

Cytokine analysis was performed by the Immune Assessment Core at UCLA using the Human Cytokine/Chemokine Magnetic Bead Panel - Premixed 38 Plex - Immunology Multiplex Assay (Millipore, Cat# HCYTMAG-60K-PX3838) following the manufacturer's instructions. 25 μL of undiluted plasma were mixed with 25 μL of magnetic beads and incubated overnight at 4°C while shaking. After washing the plate two times with wash buffer in a Biotek ELx405 washer, 25 μL of biotinylated detection antibody was added and incubated at room temperature for 1 h. 25 μL streptavidin-phycoerythrin conjugate was then added to the reaction mixture and incubated at room temperature for 30 min. Following two additional washes, beads were resuspended in sheath fluid, and fluorescence was quantified using a Luminex 200 instrument. Data was analyzed using MILLIPLEX Analyst 5.1 software.

QUANTIFICATION AND STATISTICAL ANALYSIS

Statistical analyses were performed using Prism software (GraphPad). Statistical details of experiments are listed in the figure legends. Unless otherwise indicated, statistical significance in figures is defined as: ns, not significant, $p > 0.05$; *, $p < 0.05$; **, $p < 0.01$; ***, $p < 0.001$.

Cell Reports Medicine, Volume *1*

Supplemental Information

Pharmacological Activation of Non-canonical NF-κB Signaling Activates Latent HIV-1 Reservoirs *In Vivo*

Lars Pache, Matthew D. Marsden, Peter Teriete, Alex J. Portillo, Dominik Heimann, Jocelyn T. Kim, Mohamed S.A. Soliman, Melanie Dimapasoc, Camille Carmona, Maria Celeridad, Adam M. Spivak, Vicente Planelles, Nicholas D.P. Cosford, Jerome A. Zack, and Sumit K. Chanda

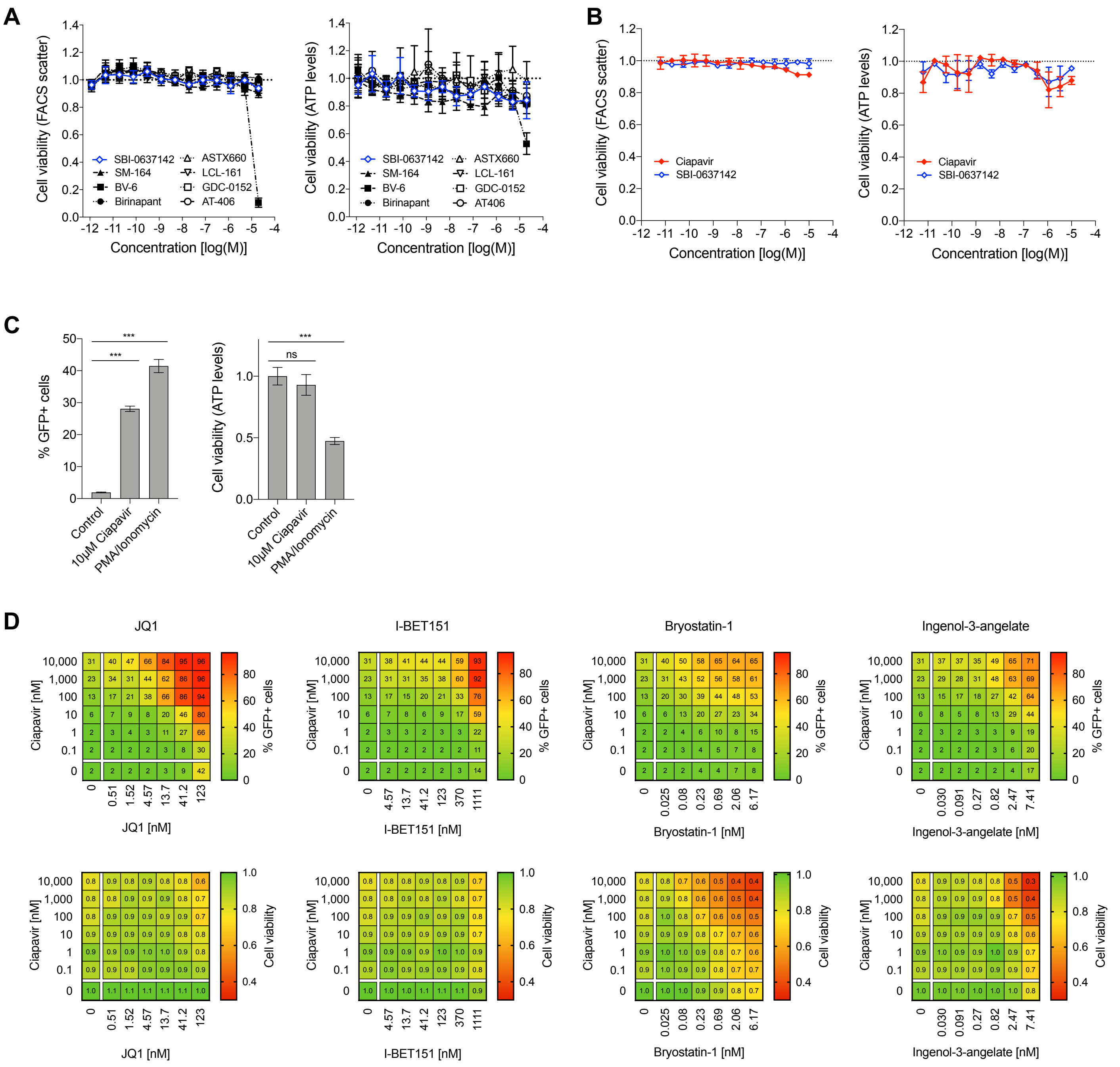

Supplemental Figure 1. Ciapavir is not toxic in 2D10 cells and synergizes with JQ1 and I-BET151. Related to Figure 1. A, B) Cell viability of 2D10 cells upon Smac mimetic treatment was assessed after 48 h by flow cytometry scatter analysis or by measuring cellular ATP levels. Data corresponds to Figures 1B and 1E, respectively, and was normalized to mock-treated samples. Data represent mean and SD (n = 2 (A); n = 3 (B)). **(C)** 2D10 cells were treated with 10 µM Ciapavir, 50 ng/ml PMA and 1 µM ionomycin, or DMSO as negative control for 48 h prior to analysis by flow cytometry. Cell viability was determined by measuring cellular ATP levels and normalized to DMSO-treated negative control. Data represent mean and SD of three biological replicates. Significance was determined with a t-test (n = 3). **D)** Ciapavir acts synergistically with JQ1 and I-BET151. 2D10 cells were treated with combinations of Ciapavir and JQ1, I-BET151, bryostatin-1, or ingenol-3-angelate for 48h and analyzed by flow cytometry for GFP expression. Cell viability was analyzed by measuring cellular ATP levels. Heatmaps show averaged data of two experiments.

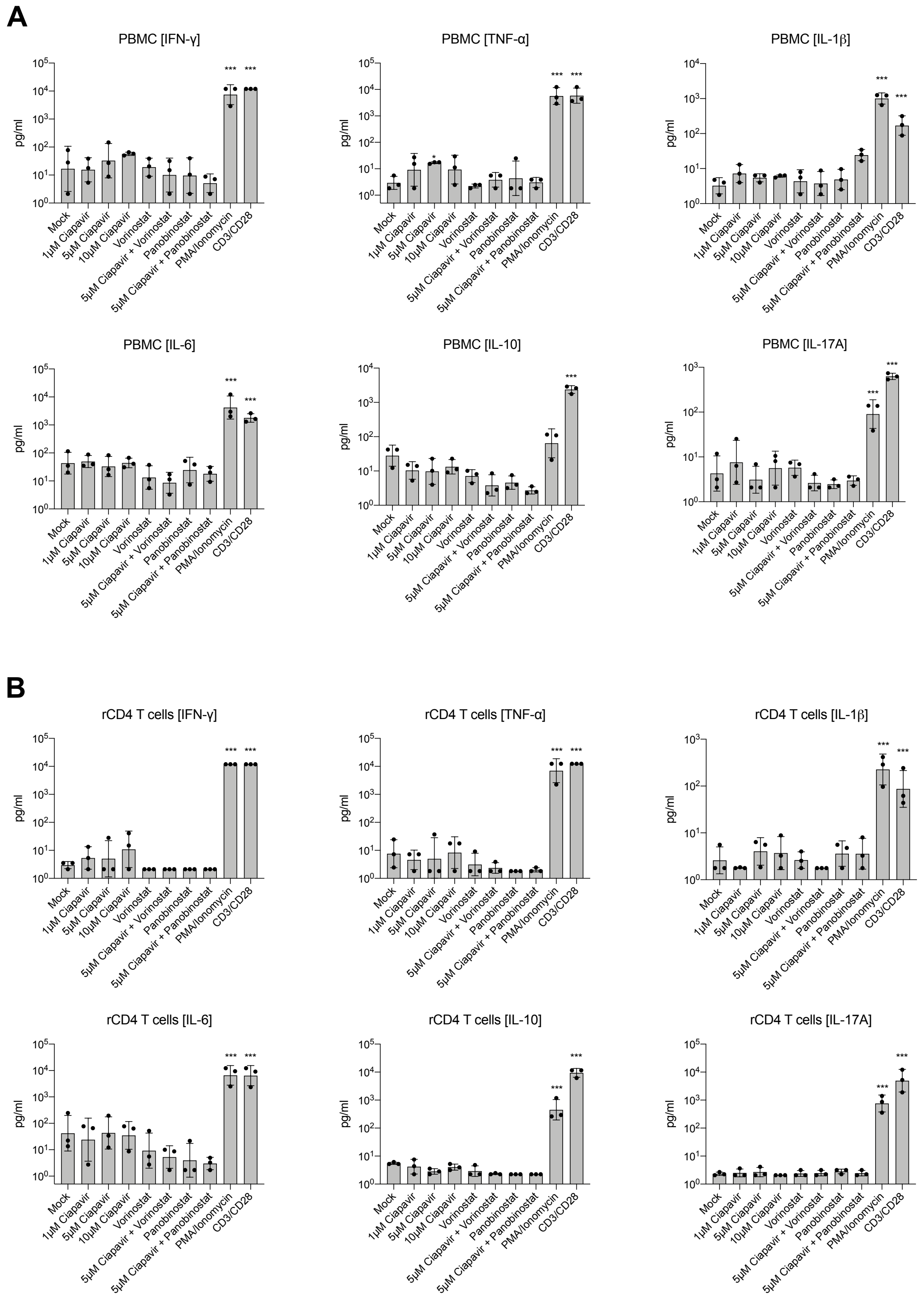

Supplemental Figure 2. Ciapavir does not induce cytokine release in human PBMC or rCD4^{+} T cells. Related to Figure 2. Values of individual donors corresponding to the data represented in Figures 2A and 2B are shown in **A)** and **B)**, respectively. Bars represent geometric mean and geometric standard deviation of three donors. To determine significance of cytokine upregulation, log transformed data was analyzed with a 2-way ANOVA using Dunnett's multiple comparison correction (n = 3).

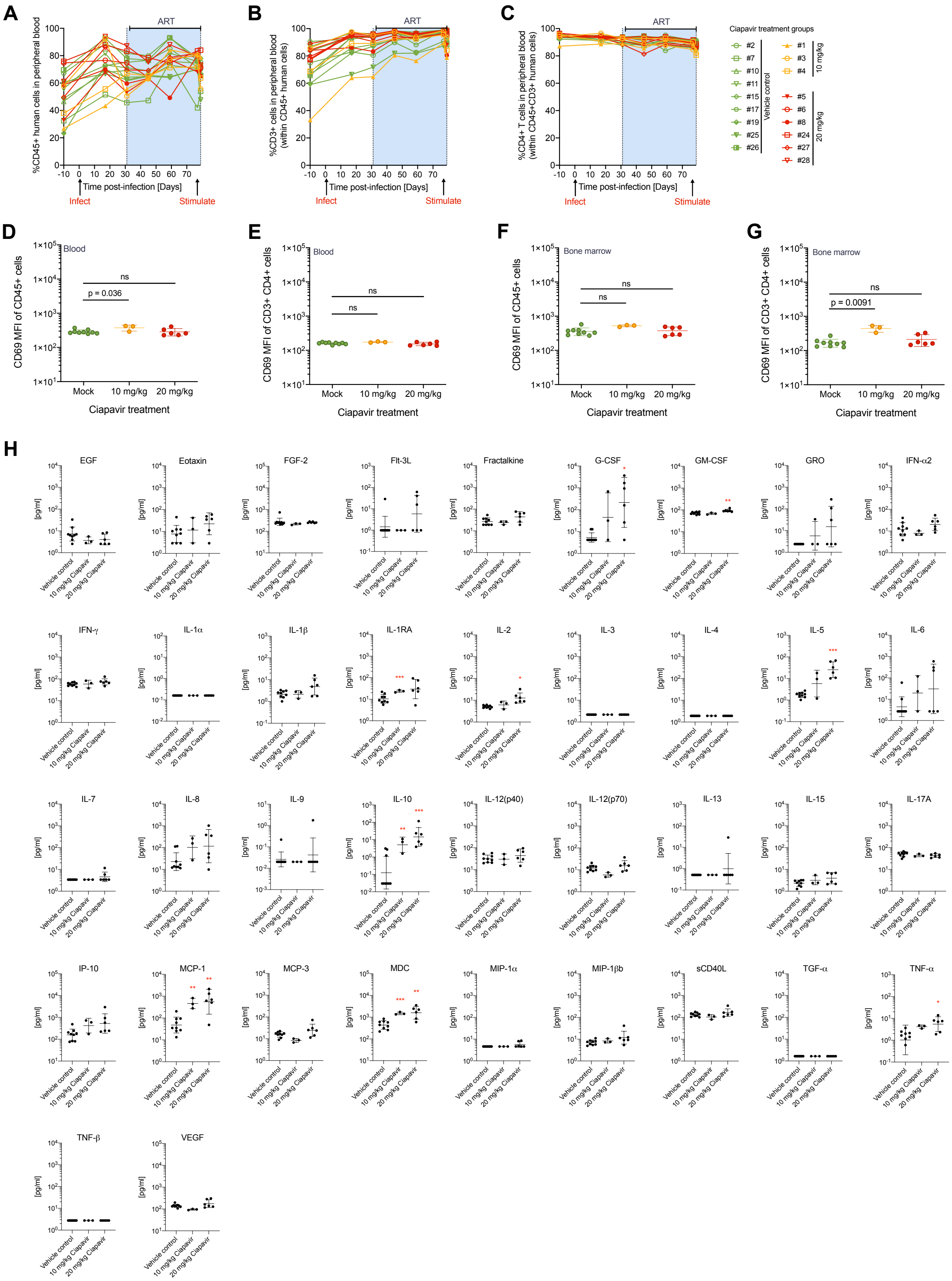

Supplemental Figure 3. Limited *in vivo* immune activation following Smac mimetic administration to humanized mice. Related to Figure 4. The frequencies of **(A)** overall human CD45$^+$ immune cells, **(B)** human T cells (CD45$^+$CD3$^+$), and **(C)** human CD4$^+$ T cells (CD45$^+$CD3$^+$CD4$^+$), analyzed by flow cytometry, did not differ significantly between treatment groups at any timepoint ($p > 0.05$, 2-sided, unpaired, equal variance t-test). Panels B and C represent frequencies of the populations from their parent gates. Mean fluorescence intensities (MFI) of samples analyzed in Figure 4E-H are shown for all blood CD45$^+$ cells **(D)** and CD4$^+$ T cells **(E)** as well as bone marrow CD45$^+$ **(F)** and CD4$^+$ T cells **(G)**. Error bars represent mean and SD (n = 9 for mock, n = 3 for 10 mg/kg Ciapavir, n = 6 for 20 mg/kg Ciapavir). Statistics represent 2-sided Mann-Whitney test. **H)** Plasma cytokine levels in BLT mice were analyzed 48 h post treatment. Data represent geometric mean and geometric SD. Significance was determined by 2-way ANOVA of log transformed data using Dunnett's multiple comparison correction (n = 9 for mock, n = 3 for 10 mg/kg Ciapavir, n = 6 for 20 mg/kg Ciapavir).

Parameter	Unit	Value
Lambda_z	1/h	0.24
$t_{1/2}$	h	2.9
T_{max}	h	0.25
C_{max}	µmol/L	8.1
T_{lag}	h	0.00
C_{last_obs}/C_{max}		0.00
$AUC_{0\text{-}t}$	µmol/L*h	10.2
$AUC_{0\text{-}inf\_obs}$	µmol/L*h	10.2
$AUC_{0\text{-}t/0\text{-}inf\_obs}$		1.0
$AUMC_{0\text{-}inf\_obs}$	µmol/L*h^2	15.8
$MRT_{0\text{-}inf\_obs}$	h	1.5
Vz/F_obs	(mg/kg)/(µmol/L)	4.1
Cl/F_obs	(mg/kg)/(µmol/L)/h	0.98

Supplemental Table 1: Detailed pharmacokinetic parameters. Related to Figure 3.
